# Chaperonin CCT controls extracellular vesicle production and cell metabolism through kinesin dynamics

**DOI:** 10.1002/jev2.12333

**Published:** 2023-06-16

**Authors:** Amelia Rojas‐Gómez, Sara G. Dosil, Francisco J. Chichón, Nieves Fernández‐Gallego, Alessia Ferrarini, Enrique Calvo, Diego Calzada‐Fraile, Silvia Requena, Joaquin Otón, Alvaro Serrano, Rocio Tarifa, Montserrat Arroyo, Andrea Sorrentino, Eva Pereiro, Jesus Vázquez, José M. Valpuesta, Francisco Sánchez‐Madrid, Noa B. Martín‐Cófreces

**Affiliations:** ^1^ Immunology Service Hospital Universitario de la Princesa, UAM, IIS‐IP Madrid Spain; ^2^ Area of Vascular Pathophysiology, Laboratory of Intercellular Communication Fundación Centro Nacional de Investigaciones Cardiovasculares‐Carlos III Madrid Spain; ^3^ Cryoelectron Microscopy Unit Centro Nacional de Biotecnología (CNB‐CSIC) Madrid Spain; ^4^ Laboratory of Cardiovascular Proteomics Fundación Centro Nacional de Investigaciones Cardiovasculares‐Carlos III Madrid Spain; ^5^ CIBER de Enfermedades Cardiovasculares (CIBERCV) Madrid Spain; ^6^ Structural Studies Division MRC Laboratory of Molecular Biology Cambridge UK; ^7^ ALBA Synchrotron Light Source Barcelona Spain; ^8^ Department of Macromolecular Structure Centro Nacional de Biotecnología (CNB‐CSIC) Madrid Spain

**Keywords:** CCT, chaperonin, extracellular vesicle, lipid droplet, lipidomic, peroxisome

## Abstract

Cell proteostasis includes gene transcription, protein translation, folding of de novo proteins, post‐translational modifications, secretion, degradation and recycling. By profiling the proteome of extracellular vesicles (EVs) from T cells, we have found the chaperonin complex CCT, involved in the correct folding of particular proteins. By limiting CCT cell‐content by siRNA, cells undergo altered lipid composition and metabolic rewiring towards a lipid‐dependent metabolism, with increased activity of peroxisomes and mitochondria. This is due to dysregulation of the dynamics of interorganelle contacts between lipid droplets, mitochondria, peroxisomes and the endolysosomal system. This process accelerates the biogenesis of multivesicular bodies leading to higher EV production through the dynamic regulation of microtubule‐based kinesin motors. These findings connect proteostasis with lipid metabolism through an unexpected role of CCT.

## SUMMARY

CCT is required to regulate interorganelle contact sites through kinesin‐1 dynamics, modulating peroxisome and mitochondria activity, and ultimately controlling lipid cell content and metabolism.

## 1 INTRODUCTION

Proteostasis is tightly regulated in cells and includes protein synthesis, folding and recycling through degradation into their building blocks, amino acids (Balch et al., 2008), as well as the release of components through sorting and secretion (Naslavsky & Caplan, 2018). To be fully functional, proteins undergo complex folding processes that can be driven by chaperones, a group of proteins that help other proteins to reach their 3D structure. Among these, the eukaryotic cytosolic chaperonin‐containing TCP1 (CCT; also called TRiC [TCP1 ring complex]) participates in the folding of a limited, number of substrates (Skjaerven et al., 2015), with new clients recently found (Cuéllar et al., 2019; Willison, 2018). CCT contains eight homologous subunits (CCT1‐8) organized into a double‐ring structure with each ring encompassing a cavity (Muñoz et al., 2011), conforming a large macromolecular complex of approximately 1 MDa (Kalisman et al., 2012; Leitner et al., 2012). The expression of actin and tubulin, major substrates for CCT, likely regulates the availability of CCT for other substrates based on their affinity and abundance (Willison, 2018). Other CCT clients belong to the WD40 family, several of which form a β‐propeller structural domain. Among these clients, mLST8 is part of mTOR complex 1 (mTORC1) (Cuéllar et al., 2019). mTORC1 regulates the flux of CCT clients by increasing the activity of eukaryotic ribosomal translation initiation factor 4E (eIF4E) binding proteins (4E‐BP1, 2 and 3), as well as p70 ribosomal S6 kinase (S6K) activity. In turn, S6K phosphorylates the S6 ribosomal subunit to initiate protein synthesis (Myers et al., 2019) and CCT2 subunit to increase its activity (Abe et al., 2009), connecting mRNA translation and protein folding during proteostasis.

Cells can also regulate their protein content through the endolysosomal system (ELS). The ELS includes early endosomes (EE), which incorporate endocytosed elements to be processed during EE maturation into late endosomes or multivesicular bodies (MVB) (Naslavsky & Caplan, 2018). MVB are organelles involved in recycling and secretion of cellular components. They contain intraluminal vesicles (ILVs) that can be formed by the ESCRT machinery, which facilitates the curvature of the MVB membrane, the incorporation of specific protein and lipidic components into the nascent ILV and the scission from the MVB membrane (Colombo et al., 2013; Kalra et al., 2012). More recently, two alternate pathways have been described, involving the synthesis of ceramide to induce vesicle curvature and budding (Trajkovic et al., 2008) and the tetraspanin‐mediated organization of specific proteins at cell membranes to sort them into ILVs (Simons & Raposo, 2009). Once formed, MVB can either converge with lysosomes (Naslavsky & Caplan, 2018) or deliver their ILVs into the extracellular medium in the form of extracellular vesicles (EVs), a class of nano‐sized biovesicles (Zaborowski et al., 2015). The study of specific components of EVs can be used to analyse the status of the producing cell and to evaluate the possible impact of these EVs on recipient cells and the extracellular matrix (Albacete‐Albacete et al., 2020). These components can be found in EV databases such as Vesiclepedia (http://microvesicles.org) and Exocarta (http://www.exocarta.org/) (Yáñez‐Mó et al., 2015).

The role of CCT has been analysed in CD4 T cells as a regulator of proteostasis during T Cell Receptor‐dependent activation by antigen‐presenting cells, which requires the translation of proteins. CD4 T cells expressing reduced levels of CCT are defective in their ability to regulate their metabolic switch upon activation, which is related to decreased mTOR activation and organelle reorganization (Martin‐Cofreces et al., 2020). T cells polarize to form the so‐called immune synapse, reorganizing the cytoskeleton and organelles such as mitochondria and lysosomes to help full T cell activation and communication with the antigen presenting cell (Martin‐Cofreces et al., 2021). This kind of cell asymmetry is relevant to transfer EVs as directional vectors from T cells to antigen‐presenting cells, since T cells re‐direct MVB to the immune synapse and release EVs there (Mittelbrunn et al., 2011). As a proof of concept, in this work we have analysed the components of EVs from different cell sources, such as mouse primary T lymphoblasts and the Jurkat E6‐1 T cell line, finding that the CCT complex is present in the released EVs together with the prefoldin complex which participates in the folding of different clients (Willison, 2018); CCT components are also described in vesiclepedia dataset (Kalra et al., 2012). However, the potential role of CCT in the regulation of the organization of organelles and the biogenesis of EVs as a consequence of deregulation of proteostasis and the consequent changes in metabolic fate of cells has not been investigated.

We have assessed the effect of limiting the availability of this chaperonin in cells, since the effect of loss of function of obligate clients may occur when CCT fails to properly assist their folding. An excess of substrates may cause unfolded forms, leading to toxicity through protein aggregation (Willison, 2018). Herein, cells with reduced expression of CCT show altered lipid composition and decreased numbers and size of lipid droplets (LDs), which establish contacts with other organelles, providing lipid components required for their biogenesis; that is: LD‐lysosome contacts promote lipid and protein transfer to lysosomes (Fransen et al., 2017). LDs also interact with mitochondria and peroxisomes to provide lipids to fuel the cell and sustain anabolic processes. We have observed that CCT regulates the dynamics of tubulin‐based molecular motor kinesin‐1, preventing proper localization of organelles and inter‐organelle contacts. Through this mechanism, CCT regulates the transfer of components from different compartments and therefore impacts the biogenesis of relevant metabolic organelles, such as peroxisomes and lysosomes, while cell viability remains unaltered. Therefore, CCT fine‐tunes the maturation of EE into MVB through the control of the lipid content in cells, with increased use of the fatty acids in the mitochondria compared with glucose, and peroxidation in peroxisomes, increasing the percentage of triacylglicerols and ceramids in cells, which promotes the formation of MVB. The final outcome of limiting CCT availability is an increased production and secretion of EVs, pointing to CCT as a connecting hub for regulation of protein degradation and secretion in cells.

## 2 MATERIALS AND METHODS

### 2.1 Antibodies and reagents

Antibodies used in this work included anti‐CD3e (HIT3a, BioLegend), anti‐CD28 (CD28.2, BD Biosciences), anti‐P150‐Glued (1/p150Glued (RUO), AB_397846, BD Biosciences), anti‐CCT1 (91a, AB_2920800, Abcam), anti‐CCT2 (5B5C4, AB_2920801, Merck Millipore), anti‐CCT4 (EPR8495(B), AB_11144306, Abcam), anti‐CCT5 (EPR7562, AB_11154964, Abcam), anti‐CD63 (NKI/C3, AB_2920802, Abcam), anti‐CD81 (5A6, AB_627192, Santa Cruz), anti‐CD63 (Tea3/18, propietary), anti‐Calnexin (AB_2069009, Abcam), anti‐EEA1 (N‐19, AB_2096822, Santa Cruz), anti‐TSG101 (4A10, AB_306450, Abcam), anti‐ADFP (EPR3713, AB_10863476, Abcam), anti‐Pex14 (AB_2736340, Invitrogen), anti‐Alpha‐Tubulin (DM1A, AB_477593, Sigma Aldrich), anti‐Alpha‐Tubulin FITC‐conjugated (DM1A, AB_476968, Sigma Aldrich), anti‐K40 alpha‐tubulin (6‐11B‐1, AB_609894, Sigma Aldrich), anti‐Kinesin Heavy Chain (H1, AB_94283, Merck Millipore), anti‐Kinesin Light Chain (L1, AB_94286, Merck Millipore), anti‐Beta‐Actin (AC‐15, AB_476692, Sigma Aldrich), anti‐TFAM (EPR12286, AB_2920803, Abcam), BODIPY™ 493/503 Neutral lipids (Invitrogen), BODIPY™ c11 581/591 Peroxized lipids (Invitrogen), LysoTracker™ Red DND‐99 (Invitrogen), MitoTracker™ Orange CMTMRos (Invitrogen), MitoTracker™ Green (Invitrogen), Nonyl‐acridine‐orange (Invitrogen), CellTracker™ Blue CMAC Dye (Invitrogen), and Phalloidin Alexa Fluor™ 647 (Invitrogen).

### 2.2 Cell culture

The Jurkat E6‐1 cell line was obtained through the NIH AIDS Reagent Program, Division of AIDS, NIAID, NIH from Dr. A. Weiss and grown in RPMI 1640 medium (Gibco‐invitrogen) supplemented with 10% fetal bovine serum (FBS, Invitrogen). HEK 293 human cell line was grown in DMEM medium (Gibco‐Ivitrogen) supplemented with 10% FBS (Hyclone). Cells were cultured at 37°C, in 5% CO_2_ atmosphere.

### 2.3 CCT silencing

E6‐1 Jurkat or HEK 293 cell lines (siCCT cells) were transfected with a pool of double‐stranded siRNAs targeting *CCT1* (5′‐CCAUUGGAGACUUGGUAAA‐3′), *CCT2* (5′‐UGGUAAACCUCGAGACAAC‐3′), *CCT4* (5′‐ AGGUGGUGCUCCAGAAAUA‐3′) and *CCT5* (5′‐CCAGACAGGUGAAGGAGAU‐3′). As a control (siCtrl cells), cells were transfected with a nonspecific siRNA (5′‐UUCUCCGAACGUGUGCACG‐3′). Cells were resuspended in Opti‐MEM (Gibco; 1 × 106 cells/mL) with 1.2 μM of each siRNA to a total of 5 μM and electroporated with Gene PulserXcell (Bio‐Rad) at 240 mV and 95 mA in 4 mm cuvettes (Bio‐Rad). Silencing was effective from 48 h until 72 h after the transfection step. HEK 293 cells were transfected with lipofectamine 2000 following the instructions of the manufacturer (Thermofisher Scientific).

### 2.4 qRT‐PCR

Transfected HEK 293 cells were collected at 48 h post‐transfection and processed following the instructions of the manufacturer to isolate RNA and perform the qRT‐PCR, as described elsewhere (Torralba et al., 2018). HPRT‐1 (Torralba et al., 2018) was used to normalize the mRNA expression of the different CCT subunits and β‐Actin (independent control); primers for CCT subunits are in Table S2.

### 2.5 EVs purification

EV fractions were obtained by differential ultracentrifugation as previously described (Théry et al., 2006). siCtrl and siCCT Jurkat E6‐1 cells (initial seeding number of 200×10^6^ cells at 10^6^ cells/mL) were cultured in RPMI medium supplemented with 10% EVs‐depleted foetal bovine serum 48 h post‐transfection (FBS, Invitrogen; FBS was depleted of bovine EVs by ultracentrifugation at 100,000 × *g* during 18 h). At 72 h, cells were centrifuged at 300 × *g* for 10 min. Later, the supernatant was centrifuged at 2000 × *g* during 20 min to remove debris and dead cells. After that, the pellet was ultracentrifuged at 10,000 × *g* for 40 min at 4°C (Beckman Coulter Avanti J‐30I, Beckman Coulter) to remove debris, apoptotic bodies, and shedding vesicles. Finally, EVs were pelleted by ultracentrifugation at 100,000 × *g* for 70 min at 4°C, washed in PBS, and collected by ultracentrifugation at 100,000 × *g* for 70 min.

### 2.6 Data from EVs produced by primary mouse lymphocytes

Data are a re‐analysis of the original proteomic datasets described in (Torralba et al., 2018) In brief, T lymphocytes were isolated and lymph nodes from spleen 6 weeks old and cultured for 48 h with 2 μg/mL concanavalin A (Sigma), washed and cultured with 50 U/mL human recombinant IL‐2 (StemCell Technologies) for at least 7 days to obtain T lymphoblasts. Then cells were washed and cultured in ultracentrifugated FCS‐ supplemented medium (10% FCS, RPMI) for 48 h, and Evs were collected as described (Théry et al., 2006) and subjected to proteomics.

### 2.7 Proteomics

Mouse samples were processed as described (Torralba et al., 2018). For Jurkat E6‐1 cell line, samples from 3 independent experiments of EV isolation from siCtrl and siCCT cells were subjected to SDS–PAGE (10% resolving gel and 4% stacking gel) at 50V. The electrophoresis was stopped when the front dye had barely passed into the resolving gel, ensuring concentration of all proteins (whole proteome) into a unique band. Staining was performed using GelCode® Blue Stain Reagent (Thermo Scientific). Gel pieces corresponding to the unique band from each of the samples were cut into cubes (1 mm). Samples were subjected to reduction with 10 mM dithiothreitol and alkylation in 50 mM iodoacetamide. For protein digestion, modified porcine trypsin (Promega) was added at a final ratio of 1:20 (trypsin‐protein). Digestion proceeded overnight at 37°C in 100 mM ammonium bicarbonate, pH 7.8. The resulting tryptic peptides were extracted by incubation in 12 mM ammonium bicarbonate (pH 8.8) followed by 0.5% TFA (trifluoroacetic acid) to a final concentration of 1%, and the peptides were lastly desalted in C18 Oasis hydrophilic‐lipophilic‐balanced (HLB) cartridges and dried‐down for further analysis.

The resulting tryptic peptide mixtures were subjected to nano‐liquid chromatography coupled to mass spectrometry for protein identification. Peptides were injected onto a C‐18 reversed phase (RP) nano‐column (75 μm I.D. and 50 cm, Acclaim PepMap100, Thermo Scientific) and analysed in a continuous acetonitrile gradient consisting of 2%–30% B in 240 min, 30%–90% B in 3 min (B = 90% acetonitrile, 0.5% acetic acid). A flow rate of 200 nL/min was used to elute peptides from the RP nano‐column to an emitter nanospray needle for real time ionization and peptide fragmentation on a Q‐Exactive HF mass spectrometer (Thermo Fisher, San José, CA). An enhanced FT‐resolution spectrum (resolution = 60,000) followed by the MS/MS spectra from most intense 15 parent ions were analysed along the chromatographic run (272 min). Dynamic exclusion was set at 30 s.

For protein identification, tandem mass spectra were extracted and charge state deconvoluted by Proteome Discoverer 1.4.0.288 (Thermo Fisher Scientific). All MS/MS samples were searched using SEQUEST (Thermo Fisher Scientific) with a fragment ion mass tolerance of 20 PPM and a parent ion tolerance of 15 PPM. Carbamidomethyl of cysteine was specified as a fixed modification. Oxidation of methionine was specified as a variable modification.

Scaffold (version Scaffold_4.2.1, Proteome Software Inc., Portland, OR) was used to validate MS/MS‐based peptide and protein identifications. Peptide identifications were accepted if they could be established at greater than 95.0% probability by the Peptide Prophet algorithm (Keller et al., 2002) with Scaffold delta‐mass correction. Protein identifications were accepted if they could be established at greater than 99.9% probability and contained at least 1 identified peptide. Protein probabilities were assigned by the Protein Prophet algorithm (Nesvizhskii et al., 2003). Proteins that contained similar peptides and could not be differentiated based on MS/MS analysis alone were grouped to satisfy the principles of parsimony.

### 2.8 Lipidomics

Five biological replicates containing 3 × 10^6^ cells were collected and stored at −80°C. Jurkat E6‐1 cell line (siCtrl and siCCT cells) was used for these experiments. The cell pellets were thawed on ice and subjected to three freeze–thaw cycles for complete cell disruption and protein precipitation. Briefly, samples were suspended in 250 μL freshly prepared methanol:acetic acid (98:2 v/v) solution, vortex‐mixed, placed in liquid nitrogen for 10s and thawed in an ice bath (for 10s) three times. Subsequently samples were centrifuged at 18,000 × *g* for 20 min at 10°C. Supernatants were collected and lipids were extracted with methyl‐tert‐butylether (MTBE) as described (Matyash et al., 2008). A total of 400 μL of organic phase were dried‐out in speedvac and resuspended in 50 μL of ACN:H2O (20:80, v:v) just before injection. Lipidomics untargeted analysis was performed using an Ultimate 3000 HPLC equipped with Agilent mRP‐Recovery C18 column (100 × 0.5 mm, 5 μm) thermostated at 55°C, coupled to an Orbitrap ELITE™ Hybrid Ion Trap‐Orbitrap Mass Spectrometer (ThermoFisher Scientific). Lipids were eluted at 100 mL/min using (A) water + 0.1% of formic acid and (B) acetonitrile with 0.1% formic acid. MS was operating in full scan mode from 70 to 1700 m/z at 60,000 resolution in positive and negative polarity mode, in separate runs.

Data processing was carried‐out using Compound Discoverer (ThermoFisher; USA) with the Metaboprofiler node (Röst et al., 2016) and MetaboAnalyst (Pang et al., 2021). Putative identification was performed using Ceu Mass Mediator (Gil‐de‐la‐Fuente et al., 2019) and our in‐house software (://github.com/CNIC‐Proteomics/TurboPutative) prioritizing match with higher probability of adduct formation and RT elution. Heatmap representation was made using MetaboAnalyst 5.0 software.

### 2.9 Soft‐X‐tomography acquisition, analysis and statistics

#### 2.9.1 CryoSXT data acquisition and image processing

Jurkat E6‐1 T cells co‐transfected with EB3‐GFP and the corresponding siRNAs (siCtrl or siCCT) were sorted for GFP 24 post‐transfection. Subsequently, GFP^+^ cells were incubated 20 min with MitoTracker Red to label mitochondria for correlative microscopy and cryo‐epifluorescent analysis of the grid as described (Martin‐Cofreces et al., 2020), with some modifications. Holey carbon‐coated (R 2/2; Quantifoil) Au‐EM finder grids were incubated with poly‐L‐lysine (50 μg/mL) for 8 h at 4°C. After that, 1.2 × 10^4^ cells were deposited on poly‐l‐lysine functionalized grids for 15 min at 37°C and cryogenically fixed by plunge‐freezing in an EM CPC vitrification unit (Leica Microsystems). From this point, grids were maintained at liquid nitrogen temperature. Vitrified grids were first analysed for screening with the cryo stage (Linkam Scientific Instruments) inserted into an AxioScope A1 (Carl Zeiss) epifluorescence microscope with an N‐Achroplan 10x/0.25 Ph1 objective. Cells were imaged with a CCD AxioCam ICm1 (Carl Zeiss). At the Mistral beamline (ALBA synchrotron (Sorrentino et al., 2015)), samples were pre‐visualized as a first stage with a 20x NA = 0.42 visible light microscope on‐line within the X‐ray microscope (Groen et al., 2019) to correlate cell position with epifluorescence and cryo‐epifluorescence images. Grid squares that contained target cells were imaged as zero‐degree soft X‐ray projection mosaics to evaluate sample conditions (vitrification and thickness). Then, cellular tilt series were acquired at 520 eV photon energy from −65° to 65° at 1° intervals, using a 25‐nm Fresnel zone plate objective lens. To avoid the depth of focus artefacts, the XTEND scheme (Otón et al., 2017) was used to acquire tilt series. In our work, the exposure time depended on sample thickness, and ranged from 1 to 4 s. The final image pixel size was 10 nm. We acquired a total of 17 XTEND data sets (1 data set = 3 multifocal tilt series). The 17 datasets from single cells corresponded to 9 siCtrl cells and 8 siCCT cells. To monitor the experimental resolution of the tomograms we used Nyquist criteria associated with the line spread function (LSF).

#### 2.9.2 SXT image quantification and statistical analysis

Automatic tilt‐series pre‐processing was performed following the previously described procedure (Otón et al., 2017). XTEND tilt series were aligned with IMOD using 150 nm Au fiducials and reconstructed with TOMO3D (Agulleiro & Fernandez, 2011) software (30 iterations of the SIRT algorithm). Semiautomatic segmentation of volumes was carried out with SuRVoS (Luengo et al., 2017), and volumes were represented with Chimera (Pettersen et al., 2004). MVB and LD statistics were calculated from segmented data. Statistical analyses were performed using in‐house python scripts (Anaconda Software Distribution; Vers. 2‐2.4.0. Anaconda, Inc., Nov. 2017. Web. http://www.anaconda.com).

### 2.10 Lipid droplet and lipid peroxidation quantification through flow cytometry

For LD quantification and lipid peroxidation, cells were stained 48 h post‐transfection with BODIPY™ 493/503 (0.8 μM; ThermoFisher) 0.8 μM BODIPY™ 581/591 C11 (ThermoFisher) in HBSS (Lonza) for 30 min at 37°C in serum‐free medium, respectively, and LIVE/DEAD™ Fixable Blue (ThermoFisher) according to manufacturer's instructions and analysed by flow cytometry. Lipid peroxidation was obtained as the MFI in the B530/30 channel (peroxidation signal with an emission wavelength of ∼510 nm) of viable cells or the fluorometric ratio between the B530/30 and YG586/15 channels (basal signal with emission wavelength of ∼ 590 nm) using a FACS Symphony SORP analyser (BD Biosciences). Flow cytometry data were analysed with FlowJo v10.8.1 software.

### 2.11 Lipid droplet imaging through confocal microscopy

Cells were stained with Lysotracker (1 nM) or Mitotracker Orange (20 nM) probes for 20 min at 37°C, washed and subsequently stained with BODIPY™ 493/503 (0.8 μM; ThermoFisher) as indicated previously, washed and allowed to adhere to fibronectin‐coated cell chambers in in vivo imaging medium (Hank's balanced salt solution (HBSS) containing 1.5% FCS and 25 mM Hepes) for 1 h before imaging. Live cells were imaged under a TCS SP5 confocal laser scanning unit fitted with an HCX PL APO lambda blue 63 × 1.40 oil objective (Leica Microsystems GmbH) using the resonant scanning rapid acquisition, with approximately 10–15 s for each Z‐scan, allowing whole‐cell recording of two wavelengths. Accompanying Application Suite X software (LAS‐AF 2.7.3. build 9723) was used. Image processing was performed with Fiji software (ImageJ; http://rsbweb.nih.gov/ij/) and with Imaris software (v9.7 Oxford Instruments) for detection of volumes and 3D reconstruction.

### 2.12 Immunofluorescence and quantification

Cells were processed as described (Blas‐Rus et al., 2017; Martin‐Cofreces et al., 2020). In brief, cells adhered to poly‐L‐Lys‐coated coverslips were fixed with 2% paraformaldehyde in PHEM (PIPES 30 mM, Hepes 20 mM, EDTA 2 mM, MgCl2 1 mM, pH: 6.9) containing 0.12 M sucrose for 10 min at R/T,permeabilized with the same solution containing Triton‐X100 (0.2%) and treated with blocking solution (PHEM containing 3% BSA, human γ‐globulin 50 μg/mL, 0.1% azide) for 30 min at R/T. Primary and fluorochrome‐conjugated secondary antibodies were incubated in blocking solution; samples were washed in Tris‐buffered saline (pH: 7.4) and mounted on Prolong Gold or Diamond mounting medium (Invitrogen). A series of fluorescence and brightfield frames were captured using a Leica SP8 Navigator Confocal Microscope equipped with a pulsed white light laser (WLL, range 470–670 nm) and an HC PL Apo CS2 100x/1.4 OIL objective and accompanying Application Suite X software (LAS X, 3.5.2. 18963; Leica Microsystems GmbH) or a TCS SP5 confocal laser scanning unit (Leica Microsystems) attached to an inverted epifluorescence microscope (DMI6000) fitted with an HCX PL APO 63x/1.40–0.6 oil objective. Images were acquired and processed with the accompanying confocal software (LCS; Leica) and Image J software (http://rsbweb.nih.gov/ij/). Quantification of tubulin acetylation was performed with Fiji and ImageJ software (Schindelin et al., 2012) to obtain the MFI relative to K40‐α‐tubulin, α‐tubulin, filamentous actin and cell area upon cell segmentation. The ratio of K40‐α‐tubulin versus α‐tubulin was calculated and normalized to the cell area. The distance from the centrosome to the peroxisomes was calculated with Imaris software, by using the Cell algorithm and the Matlab‐based algorithm to calculate the distances from surfaces to spots, by determining the localization of the centrosome and peroxisome spots and the cell surface. Centrosome‐peroxisome distance and the number of peroxisomes was normalized to the cell volume. For peroxisome quantification with anti‐Pex14, the image analysis was carried on Fiji and ImageJ software (Schindelin et al., 2012). MorpholibJ (Legland et al., 2016) and 3D ImageJ Suite (Ollion et al., 2013) libraries were installed and Java8 and ImageScience updated sites were activated in Fiji. Briefly, the image's noise was eliminated with ‘Pure denoise’ plugin (Florian Luisier at the Biomedical Imaging Group (BIG), EPFL, Switzerland; http://bigwww.epfl.ch/algorithms/denoise/). Afterwards, the background was removed with ‘Substract Background’ and objects were separated and detected with ‘3D watershed’ plugin, based on the maximum intensity. The ‘3D Manager’ tool from the 3D ImageJ Suite was employed to count peroxisomes after filtering by size.

### 2.13 ColorfulCell plasmid transfection and videomicroscopy

Jurkat E6‐1 cells were washed in HBSS (Hank's Balanced Salt Solution), resuspended in Opti‐MEM I (Invitrogen) and electroporated simultaneously with 10 μg of the ColorfulCell plasmid (MXS vector with 6 cassette inserts: CMV::GTS‐Citrine‐bGHpA, CMV::Lyn‐Ceruleanx3‐bGHpA, CMV::NLS‐TagBFPx3‐bGHpA, CMV::MTS‐ 4 AzamiGreen‐bGHpA, CMV::iRFP670‐SKL‐bGHpA, CMV::ETS‐mCherry‐KDEL‐bGHpA; https://www.addgene.org/62449/) and the corresponding control and CCT subunits siRNAs.

#### 2.13.1 Preparation of glass plates coated with anti‐CD3/anti‐CD28 antibodies

35 mm diameter plates (glass bottom, 10 mm diameter; No. 1.5 Mat‐Tek Corporation) were coated with anti‐CD3 (10 μg/mL; HIT3a clone) and anti‐CD28 (3 μg/mL; CD28.2 clone) monoclonal antibodies previously diluted in bicarbonate buffer (0.1 M NaHCO_3_, 0.32 M Na_2_CO_3_). Hundred microliters of solution were used per chamber. Before imaging, the plates were washed three times with HBSS and covered with 2 mL of imaging medium (HBSS supplemented with 1.5% FBS and 25 mM HEPES).

#### 2.13.2 Preparation of cells for imaging and sorting

Cells were washed and resuspended in 1 mL of imaging medium 48 h post‐transfection and sorted on a FACS Aria flow cytometer (BD Biosciences) 1 h before imaging. Sorted cells were maintained in 10% supplemented medium until imaging at 37°C.

#### 2.13.3 Confocal microscopy

Cells were maintained in 2 mL of imaging medium. Serial images were acquired using a Leica SP8 Navigator confocal laser unit fitted with an HCX PL APO 63x/1.40‐0.6 oil objective. 3D reconstruction was processed with the microscope‐associated confocal software (LCS; Leica).

### 2.14 Mitochondrial respiration and glycolysis assays

A XF96 extracellular flux analyser (Seahorse Bioscience; XF96 FluxPak Agilent Technologies) was used to measure OCR and ECAR.

#### 2.14.1 Glucose‐based mitostress test

The use of glucose was measured in siCtrl or siCCT Jurkat E6‐1 cells cultured with DMEM medium (D5030, Sigma Aldrich) supplemented with 1 mM sodium pyruvate, 1 mM L‐glutamine and 25 mM glucose. Cells were seeded at 0.3 × 10^6^ per well in poly‐L‐Lys pre‐coated culture plates. Each plate included five biological replicates and four technical replicates. Drugs were injected as follows: oligomycin (1 μM), CCCP (1.5 μM), rotenone (1 μM) plus antimycin A (1 μM). Three consecutive mix and measure steps were performed for resting conditions and after each injection (3 min each).

#### 2.14.2 Glycolysis assays

For glycolysis stress and rate assays, cells were cultured with DMEM medium supplemented with 2 mM L‐glutamine and seeded as before (5 biological and 4 technical replicates per plate). Injections were as follows: glucose (10 mM), oligomycin (1.5 μM), 2‐deoxyglucose (2‐DG, 50 mM) and glucose (10 mM), rotenone (1 μM) plus antimycin A (1 μM) and 2‐DG (10 mM) for stress and rate tests, respectively. Mix and measure steps were as before.

#### 2.14.3 Palmitate‐based mitostress test

To evaluate the FAO rate, siCtrl and siCCT Jurkat E6‐1 cells were cultured for in substrate‐limited growth media (DMEM medium containing 1% FCS, 2.5 mM glucose, 25 mM Hepes, 0.5 mM L‐carnitine and 1 mM L‐glutamine). The day of the experiment, cells were washed and seeded at 0.3 × 10^6^ per well in FAO assay media (111 mM NaCl, 4.7 mM KCl, 1.25 mM CaCl_2_, 2 mM MgSO_4_, 1.2 mM NaH_2_PO_4_, 1 mM glucose, 0.5 mM L‐carnitine, 5 mM Hepes, 125 μM Palmitate‐BSA) on poly‐L‐Lys pre‐coated culture plate from XF96 FluxPak (Agilent Technologies) and seeded. Drugs were injected as follows: medium or etomoxir (40 μM), oligomycin (3μM), CCCP (4 μM), rotenone (4 μM) plus antimycin A (2 μM). Three and five consecutive mix and measure steps were performed for resting conditions and after oligomycin, CCCP and roteone plus antimycin A injection (3 min each) and after etomoxir injection (3 min each), respectively. All Seahorse results were analysed with Seahorse Wave software and subjected to a linear mixed model statistical analysis (R 3.6.3 version; https://stats.idre.ucla.edu/other/mult‐pkg/introduction‐to‐linear‐mixed‐models/).

### 2.15 Nanoparticle Tracking Analysis (NTA)

EVs production was analysed in a NanoSight LM10 system. Samples were diluted in HBSS before their analysis and loaded into the sample chamber. Three videos (30 s, 1800 frames) were recorded for each sample and the tracking of particles was performed with the accompanying software using a Brownian motion‐based algorithm. Mean, median and SD were calculated for number and size distribution. Results were analysed with the help of the equipment's software.

### 2.16 SDS–PAGE

Cells were lysed in 20 mM Tris‐HCl (pH 7.5) containing 1% NP40, 0.2% Triton X‐100, 150 mM NaCl, 2 mM EDTA, 1.5 mM MgCl_2_ with phosphatase and protease inhibitors. Lysates were centrifuged at 16,000 × g and 4°C for 10 min to remove cell debris and nuclei. The resulting supernatants were diluted in Laemmli sample buffer with or without 2‐mercaptoethanol (0.15 M), and separated electrophoretically in corresponding poly‐acrylamide SDS‐PAGE. Upon transfer to nitrocellulose membranes, the extracts were subjected to western blot analyses. To this end, the filters were blocked with 5% BSA in TBS‐Tween 0.1%, incubated overnight with the primary antibodies, and the immunoreactive bands visualized upon incubation with peroxidase‐labelled secondary antibodies for 30 min. Signal detection was performed using the ImageQuant LAS‐4000 chemiluminescence and fluorescence imaging system (Fujifilm) (Blas‐Rus et al., 2017).

### 2.17 Isolation of mitochondria and subcellular fractionation

Mitochondria were isolated from siCtrl and siCCT HEK 293 cells with Miltenyi isolation kit for human mitochondria following the manufacturer instructions. The isolated mitochondria were processed in lysis buffer through sonication and subjected to immunoelectrophoresis analysis. Subcellular fractions were obtained as follows: for soluble, cytosolic proteins, cells were rinsed with 5 mL HBSS twice and scraped into the last 2 mL of HBSS, centrifuged at 2500 rpm for 3 min in a bench centrifuge at R/T. The cell pellet was then resuspended in 500 μL of lysis buffer (0.1 M MES, pH 6.7, 1 mM MgCl_2_, 1 mM EGTA, 0.1 mM GTP, 5 nM taxol, 0.2 mM dithiothreitol, 0.1 mM PMSF and protease and phosphatise inhibitor cocktail), lysed by passing it through 10 and 20 strokes with two tight pestles in a 2 mL Dounce homogenizer at R/T. An aliquot was taken and sonicated as total lysate. The rest of lysates were then centrifuged at 14,000 × *g* for 30 min at 25°C. Supernatant was collected as soluble fraction. Pellet was resuspended in 833 μL of RIPA buffer and passed through 23G, 25G and 30G needles 10 times each before centrifuge it at 14,000 × *g* for 45 min at 4°C. Supernatant was collected as the organelle fraction and pellet was resuspended in 333 μL of RIPA buffer and sonicated. Samples were processed for western blot. All fractions were run in the same gels and transferred to nitrocellulose.

### 2.18 Statistical analyses

All data were analysed with Python Software or GraphPad Prism 8.0 (GraphPad Software, San Diego, CA). Normality was assessed and parametric and non‐parametric test were used as indicated.

## 3 RESULTS

### 3.1 CCT is present in EVs

As a prospective study on relevant chaperones that maintain protein homeostasis, we performed a characterization of the proteome of EVs secreted by Jurkat E6‐1 T cell line or mouse primary T lymphoblasts. EVs were isolated by differential ultracentrifugation (Théry et al., 2006) and followed by label‐free mass spectrometry. Using this approach, we were able to identify numerous peptides corresponding to the eight subunits that conform the chaperonin CCT (Table 1). In addition, the prefoldin complex, a CCT co‐chaperone (Skjaerven et al., 2015) and the tubulin‐binding cofactors TBCA and TBCB (Willison, 2018), were found to be present in the EVs of both samples. Another chaperone found in EVs was Hsp90 (α and β isoforms) and its corresponding co‐chaperone, cdc37. We also confirmed the presence of proteins commonly enriched in MVB such as ERM (Ezrin, Radixin, Moesin), tetraspanins (CD9, CD81) and the ESCRT component TSG101 (Table 1) (Théry et al., 2018). These data indicate that several chaperones are shuttled to EVs in T lymphocytes and Jurkat lymphoblastoid cell line and suggest a relation between CCT and this MVB‐dependent secretion pathway.

**TABLE 1:** T cell‐derived extracellular vesicles contain CCT and other chaperones. Number of identified peptide sequences that conform CCT subunits, chaperones and cofactors related to CCT function and EV markers in isolated EVs from Jurkat E6‐1 cell line and mouse primary T lymphoblasts. Proteins were searched by specific classifiers and included in the table.

	Gene name	Protein description	Mouse lymphoblast	Jurkat E6.1
CCT subunits	*CCT1*	T‐complex protein 1 subunit alpha	18	14
	*CCT2*	T‐complex protein 1 subunit beta	28	16
	*CCT3*	T‐complex protein 1 subunit gamma	23	8
	*CCT4*	T‐complex protein 1 subunit delta	18	16
	*CCT5*	T‐complex protein 1 subunit epsilon	17	8
	*CCT6*	T‐complex protein 1 subunit zeta	14	3
	*CCT7*	T‐complex protein 1 subunit eta	15	12
	*CCT8*	T‐complex protein 1 subunit theta	28	20
Chaperones and cofactors related to CCT function	*HSP90AA1*	Heat shock protein 90‐alpha	35	26
	*HSP90AB1*	Heat shock protein 90‐beta	31	28
	*HSP90B1*	Endoplasmin	22	11
	*CDC37*	Cell Division Cycle 37	7	9
	*PFD1*	Prefoldin subunit 1	0	1
	*PFD2*	Prefoldin subunit 2	4	1
	*PFD3*	Prefoldin subunit 3	3	0
	*PFD4*	Prefoldin subunit 4	1	2
	*PFD5*	Prefoldin subunit 5	1	0
	*PFD6*	Prefoldin subunit 6	1	2
	*TBCA*	Tubulin‐specific chaperone A	2	2
	*TBCB*	Tubulin‐folding cofactor B	2	6
	*TBCD*	Tubulin‐specific chaperone D	5	0
	*TBCE*	Tubulin‐specific chaperone E	0	1
Extracellular vesicle markers	*CD9*	Cluster of Differentiation 9	3	1
	*CD81*	Cluster of Differentiation 81	1	2
	*EZR*	Ezrin	26	28
	*MSN*	Moesin	46	47
	*RDX*	Radixin	11	1
	*TSG101*	Tumor susceptibility gene 101 protein	12	2
	*TUBA*	Tubulin alpha	25	24
	*TUBB*	Tubulin beta	49	26
	*ACTG*	Actin, cytoplasmic 2	27	17

### 3.2 CCT maintains the cell and EV proteomic profile

**FIGURE 1:**
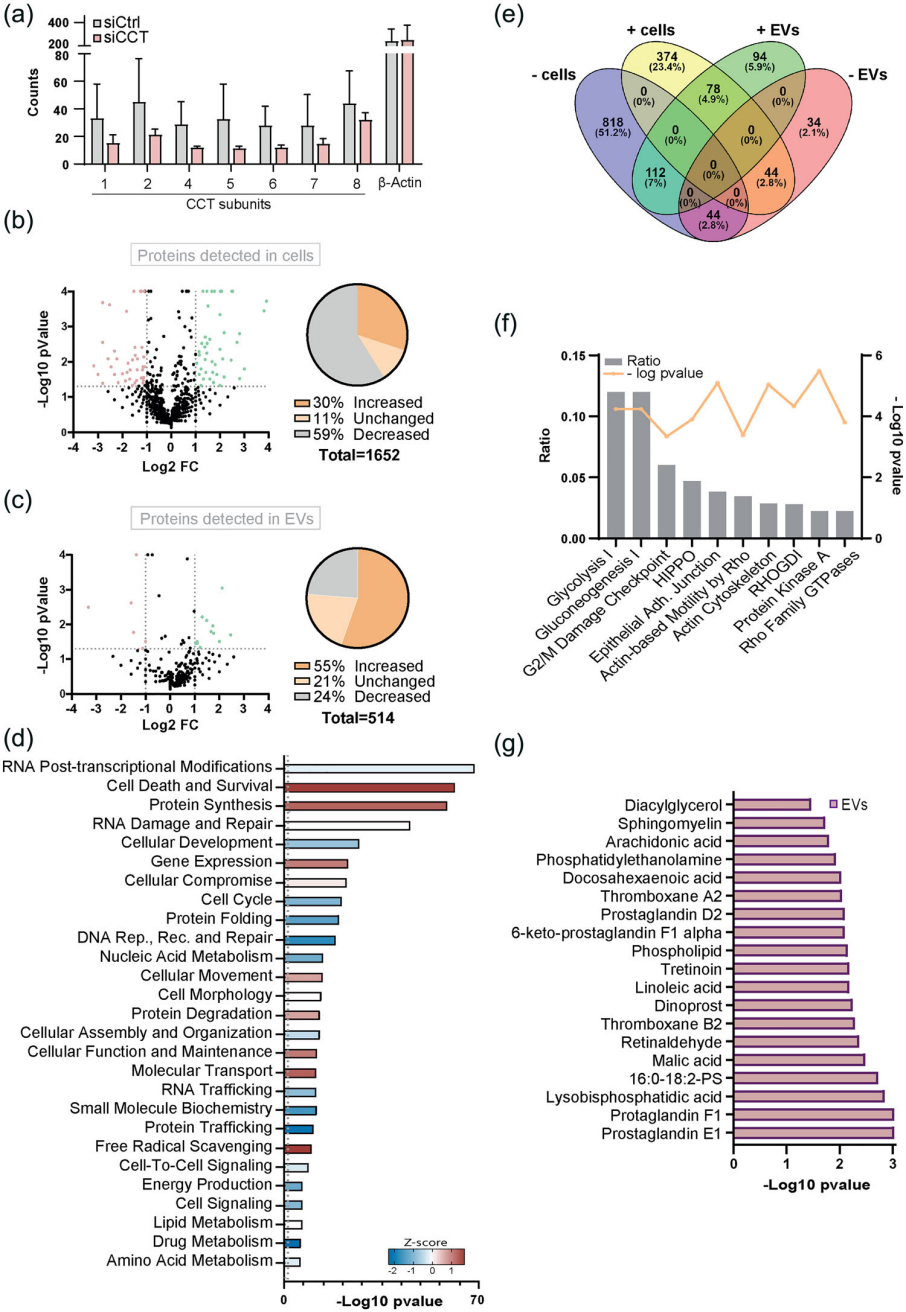
CCT regulates the proteomic profile of cells and extracellular vesicles. (a) Spectral counts of the 8 CCT subunits and the internal control beta actin recognized by mass spectrometry analysis in siCtrl and siCCT cells. Graphs, means ± SEM. (*n* = 3). (b) Volcano plot of the proteomic profile identified in siCtrl and siCCT cells (left); and pie chart representing the relative amount of proteins that are increased (dark orange), unchanged (light orange) and decreased (grey) in siCCT versus siCtrl cells (right). A total of 1652 proteins were identified. (c) Volcano plot of the proteomic profile identified in siCtrl and siCCT EVs (left); and pie chart representing the relative amount of proteins that are increased (dark orange), unchanged (light orange) and decreased (grey) in siCCT versus siCtrl‐derived EVs (right). A total of 514 proteins were identified. (d) Canonical pathways predicted by IPA related to proteins increased in siCCT versus siCtrl cells. (e) Venn diagram showing the criteria used to select the proteins that are simultaneously reduced in siCCT cells and increased in siCCT‐derived versus to siCtrl‐derived EVs (dark green). (f) Canonical pathways predicted by IPA for the proteins that are simultaneously reduced in cells and increased in EVs from siCCT condition. (g) Lipid pathways that are altered in siCCT‐derived EVs predicted by IPA. (a–f) A threshold of at least 1 peptide detected was established for this analysis. Fisher exact test was used to calculate the relative *p*‐value and fold change of the proteomic analysis (*n* = 3); cells used for this study were Jurkat E6‐1. See also Figure S1.

To investigate the potential relation between CCT and EV production, we first silenced several CCT subunits to reduce the availability of the complex by co‐transfecting Jurkat cells with either control siRNAs (siCtrl cells) or a pool of siRNAs targeting four of the CCT subunits (siCCT cells; *CCT1, CCT2, CCT4* and *CCT5*). A 53% silencing ratio was obtained for the complete target CCT set (Figure S1a). Specificity of siRNAs was corroborated by qRT‐PCR in HEK 293 cells (Figure S1b). Then, we performed a side‐by‐side proteome characterization of both cells and EVs using mass spectrometry analyses. The number of peptides corresponding to CCT subunits decreased in siCCT cells (Figure 1a), while they remained unchanged or increased in EVs (Figure S1c). We found that 975 out of the 1652 proteins detected in cells (59%) showed a decrease in siCCT cells versus siCtrl cells. We also noticed that 495 (30%) of the proteins analysed were preferentially increased in their abundance and only 182 (11%) remained unaffected in silenced CCT cells (Figure 1b). The decrease in a large proportion of proteins produced by the drop in CCT components is consistent with the described role of CCT in the folding of newly synthesized proteins (Martín‐Cófreces et al., 2021), and in preserving the stability of proteins through protein‐protein contacts (Hodeify et al., 2018). Also, some proteins were preferentially increased (283 out of 514 detected, 55%) in siCCT cell‐derived EVs (Figure 1c). These results indicate a relation between CCT, vesicular transport and EV content. By Ingenuity Pathway Analysis (IPA), we obtained clues of the main pathways that were altered in siCCT cells. We found a decrease in proteins involved in pathways related to RNA post‐transcriptional modification, DNA repair, cell cycle, protein folding and cell development, and an increase of those involved in gene expression, protein synthesis and degradation and cell death and survival. Also, cell movement and molecular transport were increased, among others (Figures 1d and S1d). Moreover, it is especially interesting that pathways related to molecular and RNA transport and to reactive oxygen species (ROS) production are increased in silenced cells, associating CCT with cell organization and metabolism (Figures 1d and S1e–f).

Next, we analysed those proteins that were reduced in cells and simultaneously increased in EVs (Figure 1e). A total of 112 proteins displaying these characteristics are involved in glucose metabolism, cytoskeletal dynamics and proliferation checkpoints (Figure 1f). In addition, IPA analysis also revealed that the proteins that are significantly augmented in siCCT‐derived EVs have a predictable role associated with lipid metabolism. This indicates that siCCT cells might use EVs as a mechanism to remove the surplus of unneeded proteins caused by the lack of CCT activity. Some important lipids related to the endolysosomal pathway predicted by IPA were diacylglycerols, sphingomyelins and phospholipids (Figure 1g). These data suggest a relationship between the chaperonin CCT and lipid homeostasis of cells.

### 3.3 CCT modulates lipid homeostasis in cells

**FIGURE 2:**
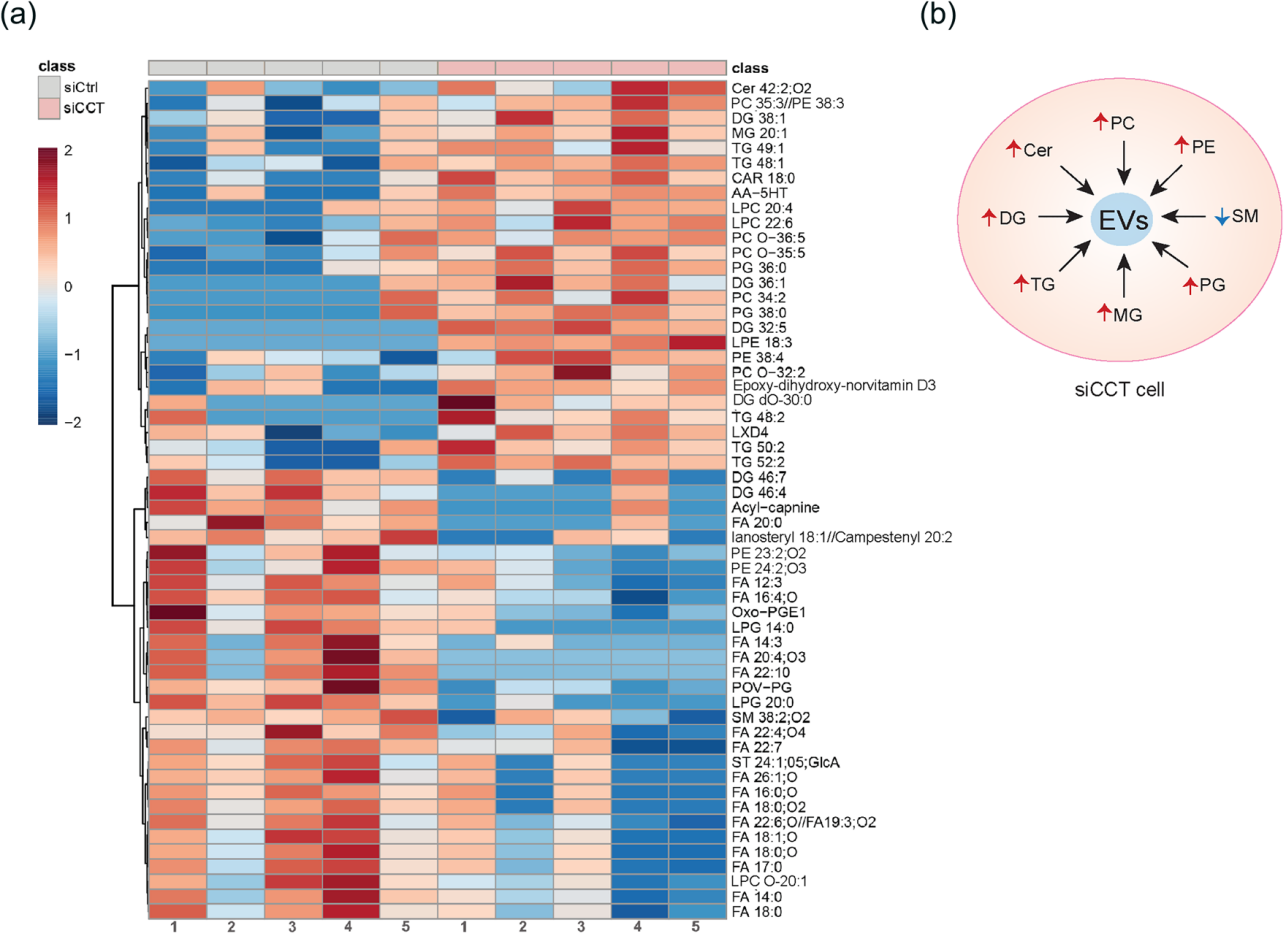
CCT is required for the maintenance of lipid profile in cells. (a) Heatmap of lipid abundance in siCtrl and siCCT cells. The figure shows the clustering results in the form of a dendrogram. Values are measured by Euclidean distance with a Ward clustering algorithm (*n* = 5 per group); cells used for this study were Jurkat E6‐1. 1–5: sample number. (b) Scheme of lipids differently present in siCCT cells, which are related to EVs biogenesis or structure. Arrows show the increase (red) or decrease (blue) of the lipid amount in the cell. Cer, ceramide; DG, diacylglycerol; MG, monoacylglycerol; PC, phosphatidylcholine; PE, phosphatidylethanolamine; PG, phosphatidylglycerol; TG, triacylglycerol; SM, sphingomyelin.

The proteomics analysis suggested that the lipid profile could be altered by CCT silencing. This prompted us to investigate possible differences in the lipid content of siCCT cells, using high throughput lipidomic approaches, when CCT is partially silenced in Jurkat E6‐1 T cell line. The study of five independent biological replicates through lipidomics for each condition evidenced different lipid content in siCtrl and siCCT cells. We analysed those lipids whose relative abundance differed significantly in siCCT cells (Figure 2a and Table S1). The group of lipids whose abundance increased in siCCT cells was mostly composed of phospholipids such as phosphatidylcholine (PC), phosphatidylethanolamine (PE) or phosphatidylglycerol (PG) and glycerolipids such as monoacylglycerol (MG), diacylglycerol (DG) or triacylglycerol (TG). Additionally, there was an increase of the bioactive lipid Ceramide (Cer) in siCCT cells (Figure 2a and Table S1). Cer, PC, PE and PG are well‐known lipids related with MVB structure and biogenesis (Skotland et al., 2017; Subra et al., 2007) (Figure 2b). In contrast, fatty acids (FAs) were decreased in siCCT cells (Figure 2a and Table S1). FAs are used by mitochondria and peroxisomes as substrates for fatty acid β‐oxidation (FAO) to obtain energy. A lipid that increases its presence in siCCT is acylcarnitine (CAR 18:0; Figure 2a and Table S1), which is used to shuttle FAs into the mitochondria matrix, and suggests a possible use of FAs in siCCT to preferentially fuel the mitochondria, pointing to an unexpected function of CCT in the regulation of the lipid metabolism.

### 3.4 CCT regulates the number and location of LDs

**FIGURE 3:**
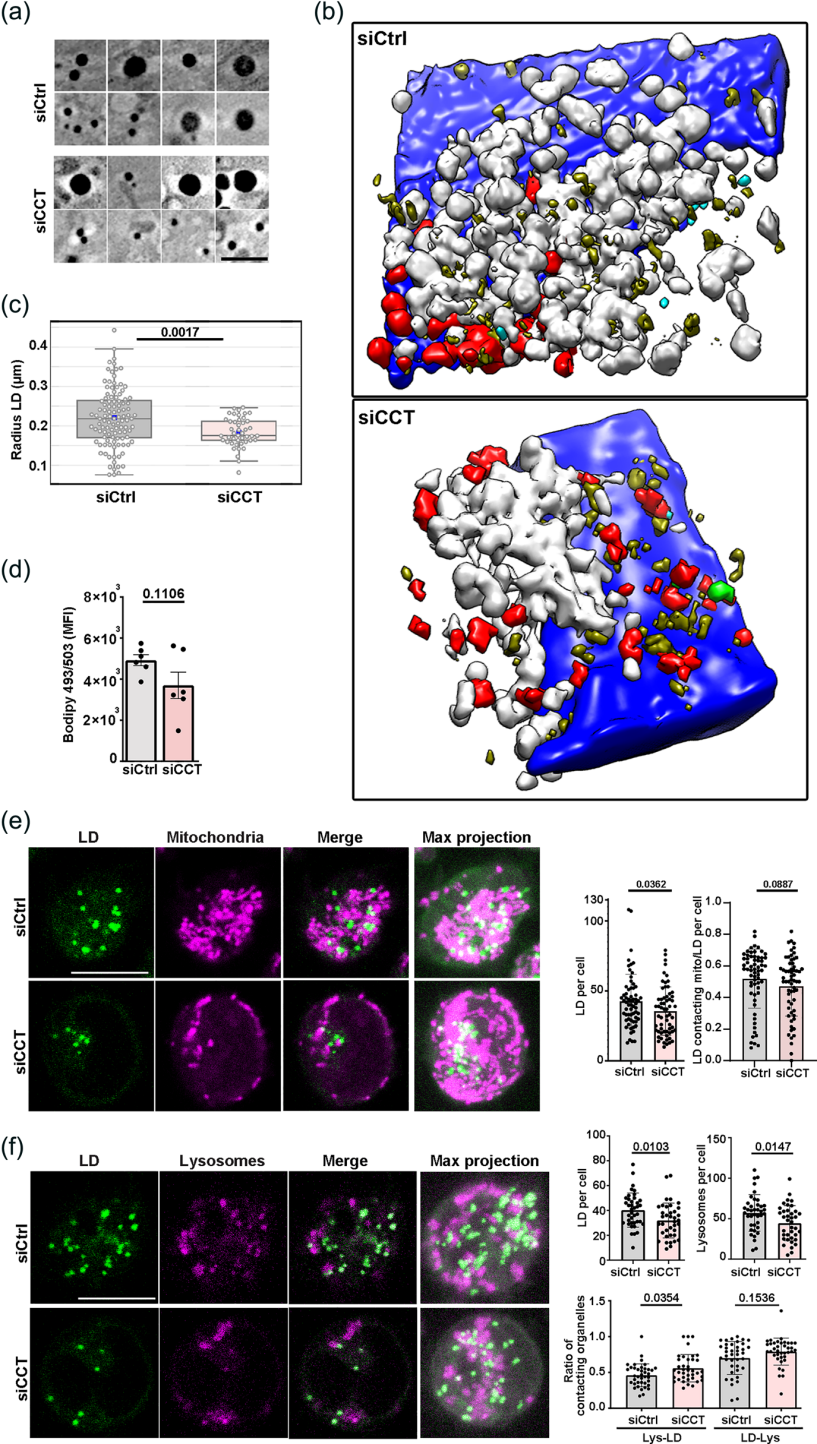
CCT is required for correct lipid droplet internal structures. (a) CryoSXT virtual slices of LDs observed as black vesicles. Bar, 200 nm. (b) 3D segmentation of the reconstructed cryoSXT volume showing MVB (white), LDs (olive green), mitochondria (red), centrioles (light green) and nucleus (blue). Field of view, 100 μm^2^. (c) Quantification of cryoSXT imaging data from (a) and (b) (mean ± interquartile range; Kruskal‐Wallis test; siCtrl *n* = 108 [8 tomograms], siCCT *n* = 48 [6 tomograms]). (d) Neutral lipids were analysed by FACS with the Bodipy 493/503 lipid probe (mean ± SEM; two‐tailed unpaired t‐student test; *n* = 6, representative of 3 independent experiments). (e) Images of LDs (green; Bodipy 493/503) and mitochondria (magenta; Mitotracker Orange) in live cells. Bar, 10 μm. Left graph, LDs per cell. Right graph, LDs contacting mitochondria per cell (estimated distance 0.5 μm; mean ± SD; Mann‐Whitney test; siCtrl *n* = 63, siCCT *n* = 62). (f) Images of LDs (green) and lysosomes (magenta; Lysotracker Red) in live cells. Bar, 10 μm. Upper graphs show the number of LD or lysosomes per cell, lower graph shows the ratio of lysosomes contacting LDs and LDs contacting lysosomes per cell (estimated distance 1.2 μm; estimated average diameter of LDs and lysosomes were 0.5 and 0.750 μm, respectively; mean ± SD; Welch's *t*‐Test; siCtrl *n* = 47, siCCT *n* = 48); cells used for this study were Jurkat E6‐1. See also Figure S2.

To ascertain whether CCT regulates the lipid metabolism in cells in terms of content and use of FAs, we studied the lipid droplets (LD) number and composition. LDs are organelles formed by a single layer of phospholipids that surround a core of neutral lipids, are closely related with lipid storage and metabolism and arise from the endoplasmic reticulum (ER). Different lipids are present within LDs, such as free fatty acids, although TG and cholesterol esters predominate and are responsible for LD structure (Olzmann & Carvalho, 2019). Therefore, we investigated the state of these organelles in silenced CCT cells by imaging vitrified siCtrl and siCCT Jurkat cells using cryo‐soft‐X‐rays tomography (cryoSXT). SXT is a non‐invasive cell imaging method that avoids the modification of cell membranes and lipids caused by fixation or the use of chemical agents (Jamme et al., 2020), and allows 3D reconstruction of thick specimens (up to 10 μm) in the 50‐nm resolution range (Otón et al., 2016). We obtained images with a pixel size of 20 nm. In these images, LDs are observed as dark vesicles due to their strong absorbance of X‐rays (Figures 3a and S2). The 3D reconstruction of tomograms showed that the localization of LDs (olive green) relative to other organelles, such as mitochondria (red) and MVB (white) was altered in siCCT cells (Figure 3b and Videos S1 and S2). The posterior reconstruction and quantification of the LDs showed that their radius decreased in siCCT cells (fold reduction of 1.20, with a median of 183.25 nm in siCCT cells vs. 219.69 nm in siCtrl cells; Figure 3c). Since SXT did not allow assessment of whole cells at the required magnification due to their dimensions and the number of tomograms needed, we analysed the cell content in neutral lipids by flow cytometry, which shows a tendency to decrease, although not significant in siCCT cells (Figure 3d). We also studied the localization and number of LDs in live cells through confocal microscopy in the context of contacts with other organelles (Figure 3e,f), such as mitochondria and lysosomes, which are known to establish contacts with LDs (Olzmann & Carvalho, 2019). The LD‐lysosome contacts facilitate lipid and protein transfer to lysosomes without the requirement of autophagosome formation or their enclosure by a double membrane organelle (Schulze et al., 2020). The analysis of LDs showed that both their number per cell (Figure 3e,f), and the number of contacts established with mitochondria (Figure 3e) were decreased in siCCT cells, whereas the percentage of lysosomes contacting with LDs increased in these cells (Figure 3f). However, the number of lysosomes detected was lower in siCCT cells (Figure 3f). Increased contacts between lysosomes and LDs may indicate that LD components are more likely incorporated into lysosomes, where neutral lipids like TGs are transformed into FAs. These FAs can be incorporated into other LDs or vesicles and transferred to different organelles (Yang & Wang, 2021), being used as structural elements for biogenesis of membrane organelles or as substrates for catabolic reactions. Since contacts between LDs and mitochondria are less frequent in siCCT cells and peroxisomes are also involved in FAO, these could be candidate organelles to examine in siCCT cells.

### 3.5 CCT regulates the fate of the peroxisome system

**FIGURE 4:**
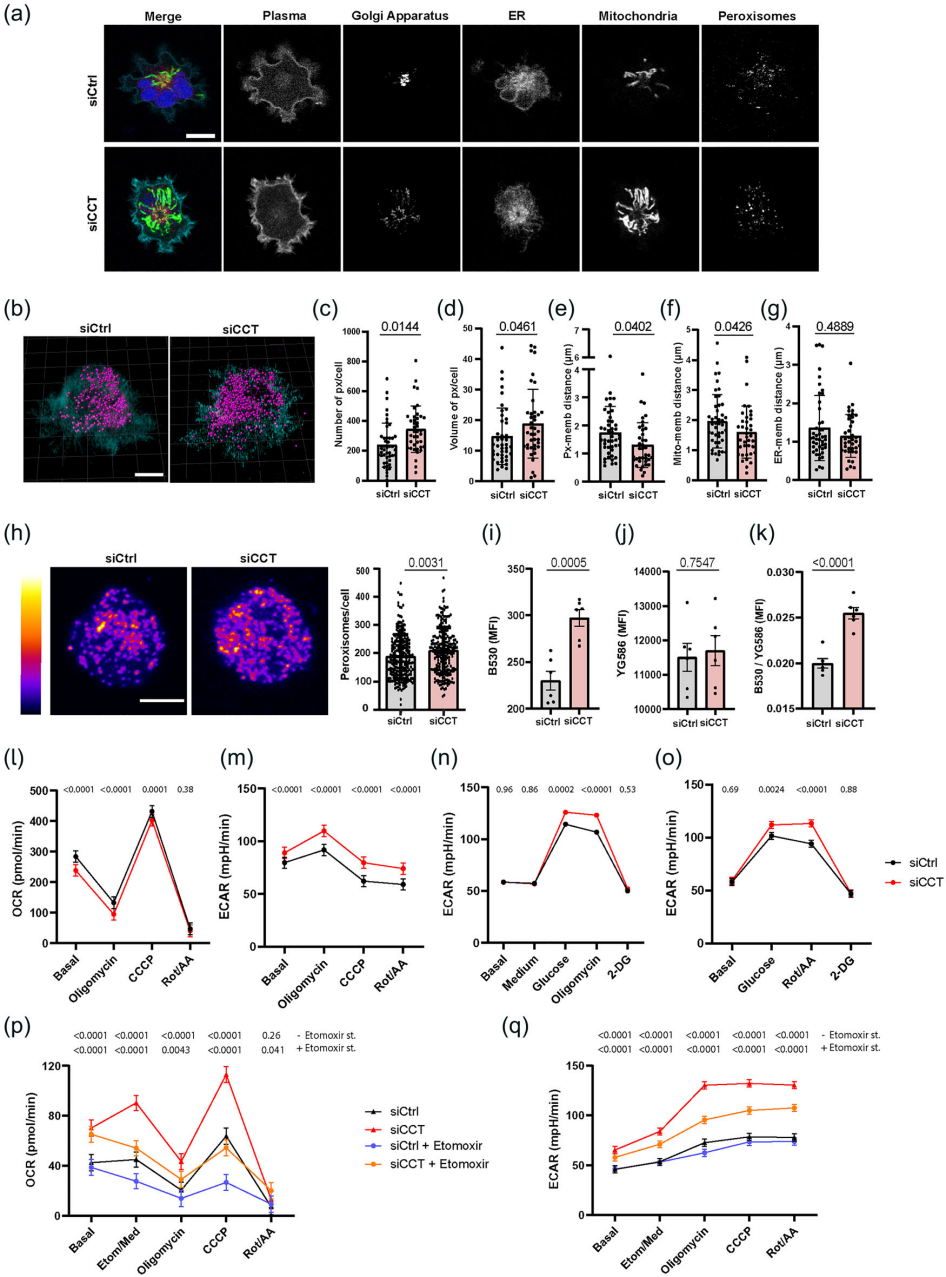
CCT regulates the number of peroxisomes and energy metabolism in cells. (a) Organelle distribution in live siCtrl and siCCT cells spreading onto stimulating anti‐CD3 and anti‐CD28 antibodies coated glass‐bottom chambers; a single plane from confocal volumes is shown. Merge: yellow, Golgi apparatus; cyan, membrane; blue, nucleus; green, mitochondria; magenta, peroxisomes; red, ER. Bar, 10 μm. (b) 3D‐reconstruction of siCtrl and siCCT cells (magenta, peroxisomes; cyan, membrane). Bar, 5 μm. (c–d) Number (c) and volume (d) of peroxisomes per cell. (e–g) Graphs showing the distance from plasma membrane to the peroxisomes (e), the mitochondria (f) or the ER (g) (mean ± SEM; two‐tailed *t*‐Student test; siCtrl *n* = 45, siCCT *n* = 40). (h) Peroxisome distribution in fixed cells was assessed with Pex‐14 antibody in resting Jurkat E6‐1 cells. Images are the sum of slices from a confocal volume. Bar, 5 μm. MFI (0‐255); Graph, peroxisomes per cell (mean ± SD; Mann Withney Test; siCtrl *n* = 236, siCCT *n* = 226). (i–k) Lipid peroxidation by flow cytometry (BODIPY™ 581/591 C11 probe): (i) peroxidation signal, B530/30 channel; (j) basal staining, YG586/15 channel; (k) fluorometric ratio (B530/30:YG586/15). Mean ± SEM; two‐tailed t‐Student test; *n* = 6, representative of 3 independent experiments. (l–m) OCR (l) and ECAR (m) in siCtrl and siCCT cells fed with 25 mM glucose, sodium piruvate and glutamine (1 mM each) and subjected to mitostress test. (n–o) ECAR from siCtrl and siCCT cells fed with glucose 10 mM and subjected to glycolysis stress test (n) or glycolytic rate assay (o). (p–q) OCR (p) and ECAR (q) in siCtrl and siCCT cells fed with palmitate (16:0; 125 μM) and treated or not with 40 μM etomoxir after basal measurements. (l–q), linear mixed model; (*n* = 5, 4 technical replicates each; from 2 (glucose mitostress test), 1 (glycolysis stress test and glycolytic rate assay) and 3 (palmitate mitostress test) experiments; graphs show means of 3 or 5 (etomoxir) serial measures; treatments are indicated (X‐axis). Px: peroxisome. Memb: membrane. Mito: mitochondria; cells used for this figure were Jurkat E6‐1. See also Figures S3 and S4.

Together, our lipidomic, cryoSXT and optical microscopy data showed a considerable modification of the lipid profile of siCCT cells, including metabolic‐related organelles such as LDs. Thus, we assessed the distribution of LD‐related cell organelles such as peroxisomes, ER and mitochondria in whole siCCT cells. Cells were co‐transfected simultaneously with control or CCT siRNAs and the ColorfulCell reporter plasmid, expressing six fluorescent proteins localized in the Golgi apparatus (Citrine), membranes (Cerulean), nucleus (TagBFP), mitochondria (AzamiGreen), peroxisomes (iRFP670) and ER (mCherry) (Sladitschek & Neveu, 2015). Images were acquired in real time during cell spreading onto stimulating anti‐CD3 and anti‐CD28 antibodies‐coated glass‐bottom chambers to analyse the dynamic organization of the intracellular compartments (Martin‐Cofreces et al., 2021) (Figure 4a–g). Interestingly, peroxisomes were larger and more abundant in siCCT cells (Figure 4b–d; 1.44 fold in number (means 238.1 vs. 344.7; medians 206.0 vs. 330.0 in siCCT cells) and 1.28 fold larger in siCCT cells (means 14.69 vs. 18.84; medians: 11.75 vs. 16.10 μm^3^). The distance from the plasma membrane to peroxisomes or mitochondria was decreased in siCCT cells, while the distance to ER remained unchanged (Figure 4e–g). Peroxisomes tend to co‐localize with the ER and mitochondria in both siCCT and siCtrl cells (Figure S3a–b). The nuclei, Golgi apparatus and ER did not display any apparent alteration under the same experimental conditions. The cell volume, area and sphericity also remained unaffected (Figure S3c). We corroborated that the number of peroxisomes was increased in resting siCCT cells by addressing the distribution of endogenous peroxisome biogenesis protein 14 (Pex14) (Figure 4h; mean 187.4 vs. 209.3, median of 175.5 and 208.5, the minimum number of particles detected were 18 and 47 and the maximum 448 and 467 for siCtrl and siCCT cells, respectively; 1.16 or 1.18 fold increase). Pex14 is an essential component of the peroxisomal import shuttle that regulates its motility along microtubules (Bharti et al., 2011; Fransen et al., 1998). Therefore, these data show a simultaneous decrease of LD volume and an increment in peroxisome number and volume in siCCT cells, either in resting or activated conditions (Figure 4b–d,h). Moreover, our lipidomic analysis evidences a significant decrease in the content of several FAs in siCCT cells (Figure 2). Accordingly, the reduced availability of CCT could trigger a rise of the FAO rate in peroxisomes and a reduction of FAs in LDs. To assess this possibility, we studied the peroxidation of lipids in siCCT cells by using a specific probe for neutral lipids that changes its emission wavelength from ∼590 nm (YG586) to ∼510 nm (B530) when lipids are peroxidised. The ratio of peroxidised lipids was increased in siCCT cells (Figure 4i–k). These results suggest that peroxisomes actively catabolize FAs in siCCT cells leading to the observed decrease of FAs. Since acylcarnitine was increased in siCCT cells and it is used by the peroxisomes to shuttle FAs to the mitochondria, we studied the metabolic fate of these cells through Seahorse XF mitostress test (Figure 4l,m). Our results showed that the oxygen consumption rate (OCR) was lower in siCCT cells (Figure 4l), whereas the extracellular acidification rate (ECAR), a measure of glycolysis, was higher (Figure 4m). We performed two different assays to test the glycolytic capacity of siCCT cells by feeding the cells with glucose (acute injection) and inhibiting the F0/F1 ATPase with oligomycin or the mitochondrial complex I and III with rotenone (Rot) and antimycin A (AA), respectively. We found that these cells preferentially used glycolysis rather than mitochondrial respiration to completely catabolise glucose (Figure 4n,o, Figure S4a‐b). We then fed the cells with FAs as energy source by supplementing with palmitate (16:0). siCCT cells showed increased OCR and ECAR when palmitate was the main substrate (Figure 4p,q). This result was corroborated by using the carnitine palmitoyltransferase I (CPT1) inhibitor etomoxir. This inhibitor clearly diminished both OCR and ECAR of siCtrl and siCCT cells, although siCCT cells still showed higher OCR than siCtrl cells. This effect may be due to the increased acylcarnitine content in siCCT cells. These data support that siCCT cells shift the energetic profile towards a lipid‐dependent metabolism.

### 3.6 CCT regulates the endolysosomal system

**FIGURE 5:**
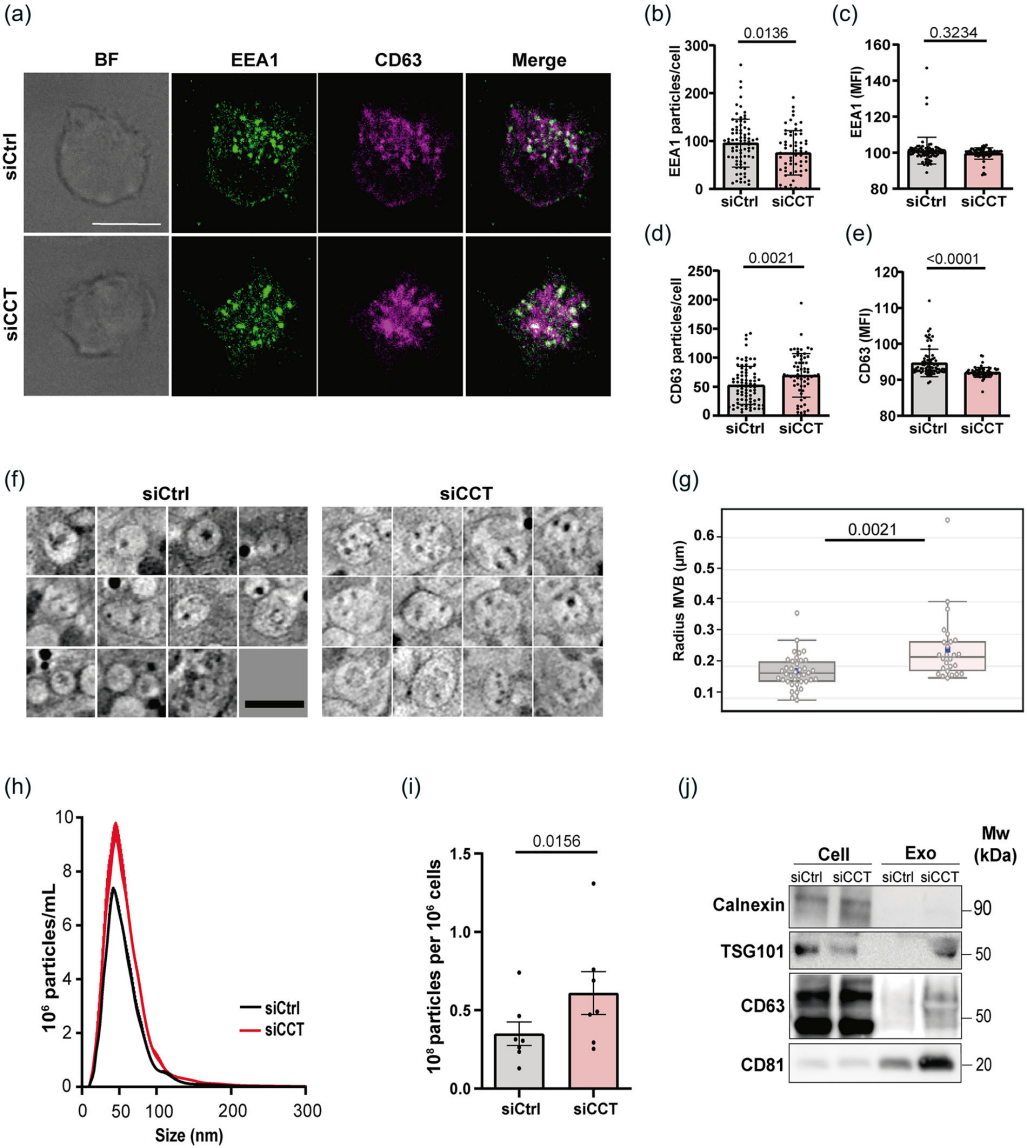
CCT controls the biogenesis of MVB and EVs. (a) Imaging of siCtrl and siCCT Jurkat E6‐1 cells. Projections of XY stacks for single bright‐field (BF) and fluorescence images are shown. Merge shows EEA1 (green) and CD63 (magenta). Bar, 10 μm. (b and c) Statistical analysis of EEA1+ particles. (d and e) Statistical analysis of CD63+ particles. (a–e) Mean ± SEM; Mann Whitney test; siCtrl *n* = 64, siCCT *n* = 81). (f) CryoSXT virtual slices of MVB. Bar, 200 nm. (g) Statistical analysis of MVB mean radius from the reconstructed tomograms. Median ± interquartile range; Kruskal‐Wallis Test; siCtrl *n* = 38 (8 tomograms), siCCT *n* = 26 (6 tomograms). (h) Size distribution analysis by NTA of purified EVs from siCtrl (black) and siCCT (red) cells (representative graph of *n* = 3 independent experiments). (i) EV production ratio per 10^6^ cells obtained from NTA quantification (Mean ± SEM; Wilcoxon *t*‐test; *n* = 7). (j) Western blot analysis of EVs purified fraction. Cells and EVs were blotted for the ER‐specific protein calnexin and for the EV markers TSG101, CD63 and CD81 (representative image of at least 3 independent experiments). Cells used for this study were Jurkat E6‐1.

We next investigated the possible role of CCT in regulating the biogenesis of ELS components, since our lipidomic analysis showed an increase of lipids that are involved in MVB structure and biogenesis (Figure 2). The ELS is a dynamic compartment that initiates with the organization of the EE, which transit towards late endosomes as MVB containing ILVs. Early endosome antigen 1 (EEA1) is an exclusive protein of EE, whereas CD63 is a common marker of ILVs (Colombo et al., 2014; Kalra et al., 2012; Mathieu et al., 2019). The simultaneous analysis of both proteins provides a suitable perspective of intracellular vesicle transport (Figure 5a). Image analysis revealed that the relative amount of EEA1 particles was significantly decreased (Figure 5b) while the mean fluorescence intensity (MFI) remained unaffected (Figure 5c). Conversely, the number of CD63 particles detected was increased in silenced cells (Figure 5d), while the MFI per particle decreased (Figure 5e), indicating that CD63‐enriched organelles were more abundant in siCCT cells. The MVB status was further studied by an exhaustive analysis of vesicular components identified in the cryoSXT slices (Figure 5f). Such slices revealed a considerable increment in the MVB mean radius in siCCT cells (Figure 5g). These findings, together with the increase of EV‐related lipids such as Cer, PC, PE and PG found in siCCT cells (Figure 2b), led us to assess EV production and release. Both siCtrl and siCCT cells were incubated for 24 h to allow EV release to the culture medium. Isolated EVs were analysed using both western blot (WB) and nanoparticle tracking analysis (NTA). The estimated average diameter of the EVs using NTA ranged from 50 to 150 nm in both siCtrl and siCCT cells, ensuring the EV nature of the samples (Figure 5h). siCCT cells secreted higher amount of EVs (Figure 5i). Also, EVs were recognized by WB based on the presence of two tetraspanins (CD63 and CD81) and an ESCRT family member (TSG101), three well known markers of these vesicles (Théry et al., 2018) (Figure 5j). Indeed, the absence of the specific ER protein calnexin ruled out the presence of cellular debris in the purified fraction (Figure 5j). WB analysis demonstrated that EV specific markers were particularly enriched in EVs from siCCT cells (Figure 5j), evidencing the increment of EV production when CCT is silenced. Taken together, these data indicate that CCT plays an essential role in EV synthesis and release, redirecting the endolysosomal pathway to MVB biogenesis and EV release.

### 3.7 CCT tunes the dynamics of kinesin‐1

**FIGURE 6:**
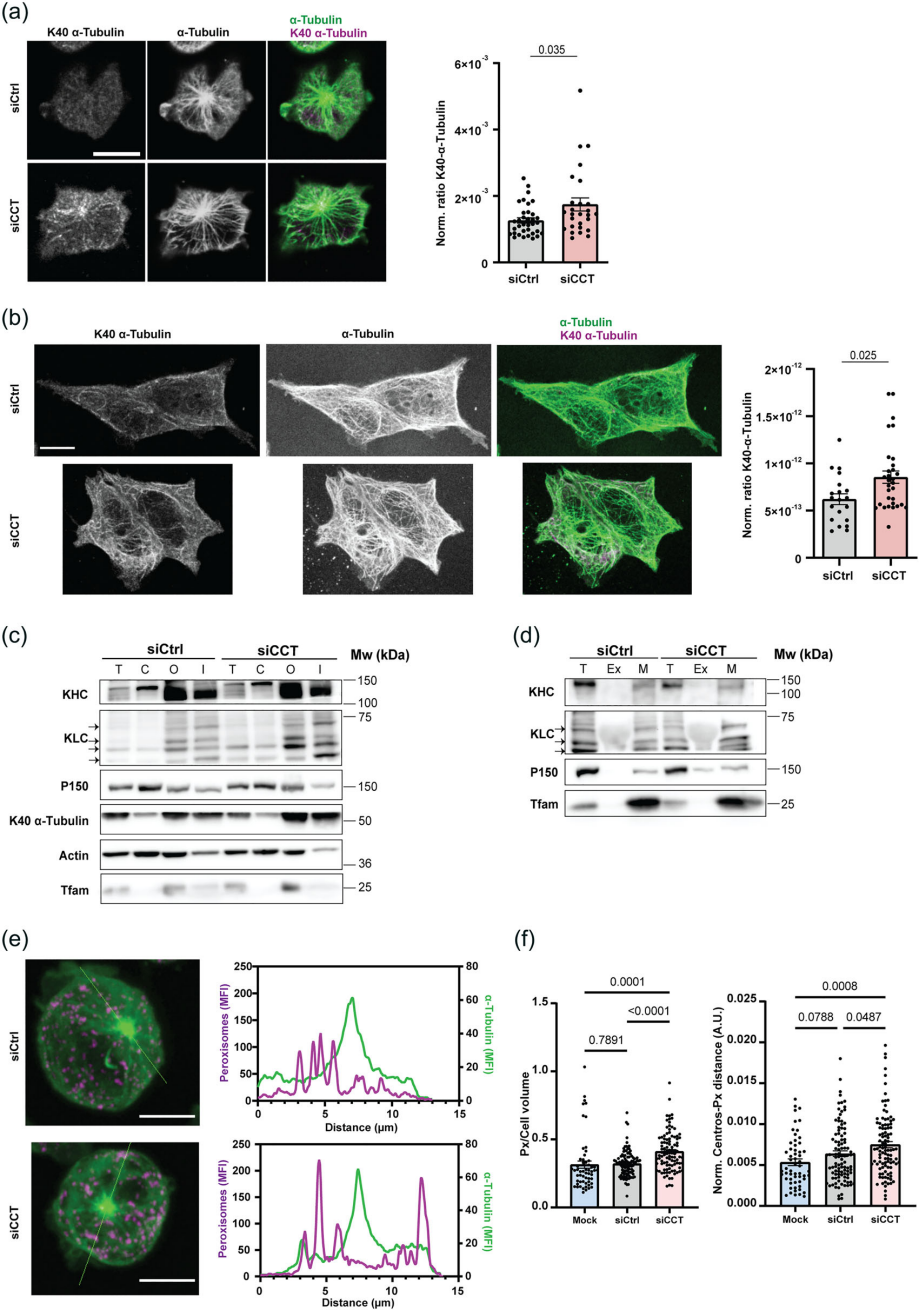
CCT regulates inter‐organellar contacts through the cytoskeleton. (a and b) Tubulin cytoskeleton of siCtrl and siCCT Jurkat cells (a) and HEK 293 cells (b). A single slice from XYZ confocal stacks containing the centrosome is shown. Merge shows α‐tubulin (green) and acetylated tubulin (K40 α‐tubulin; magenta). Graphs, normalized acetylated (K40) α‐Tubulin:α‐Tubulin ratio (Mean ± SEM; Mann Whitney test; (a) siCtrl *n* = 36, siCCT *n* = 26 and (b) siCtrl *n* = 20, siCCT *n* = 31). Bar, 10 μm. (c and d) Western blot showing distribution of indicated proteins in (c) subcellular fractions (T, total extract; C, cytosol; O, organelles; I, insoluble) and in (d) isolated mitochondria (M) from siCtrl and siCCT HEK 293 cells (T, total; Ex, excluded volume from the columns) (representative image of three independent experiments). (e) Images, Peroxisomes (magenta) and α‐tubulin (green) location in siCtrl and siCCT Jurkat E6‐1 cells. A maximal projection is shown. Plots, MFI and location of the signal in siCtrl and siCCT cells. (f) Graphs, normalized peroxisome number and distance from centrosome to peroxisomes per cell volume in mock, siCtrl and siCCT cells (mock, *n* = 54, siCtrl *n* = 101, siCCT *n* = 102). Graph, mean ± SEM. One way Anova, KHC, kinesin heavy chain; KLC, kinesin light chain. See also Figure S5.

The intracellular number and quality of contacts among organelles is essential to regulate their shape and functions (Klecker et al., 2014; Scorrano et al., 2019; Vance, 2020). This specific organization may be achieved through the control of their movement and docking. These events are dependent on different molecular motors, such as dynein and kinesins, and the microtubule cytoskeleton, which serves as rails and docking sites (Hancock, 2014). These motors regulate the retrograde and anterograde transport of cargoes in the cell. Hence, dynein mostly moves towards the minus‐end of microtubules and the majority of kinesins drive cargoes towards the plus‐end of microtubules (i.e., centrosome and plasma membrane, respectively) (Hancock, 2014). The ability of these motors to bind to the cytoskeleton depends on different factors, such as tubulin post‐translational modifications (Hancock, 2014). We therefore analysed the organization of microtubules in siCtrl and siCCT cells, and observed that the acetylation of the lysine 40 in α‐tubulin (K40 α‐tubulin) was increased in siCCT cells, both in Jurkat E6‐1 and HEK 293 cells (Figure 6a,b). This was accompanied by the formation of microtubule bundles, visualized as thicker filaments of αβ‐tubulin. Since kinesin‐1 binds preferentially to acetylated microtubules due to their tendency to form bundles (Balabanian et al., 2017), we assessed the presence of this motor protein in cytoskeleton fractions from siCtrl and siCCT cells (Figure 6c). Kinesin‐1, a complex formed by kinesin heavy chain (KHC) and kinesin light chain (KLC), was enriched in insoluble cytoskeletal fractions (I) of siCCT cells (Figure 6c). K40‐α‐tubulin was also more abundant in this fraction in siCCT cells. The adaptor dynactin (p150 subunit is shown; Figure 6c), which helps kinesin and dynein in the binding to different organelles, was unaffected in siCCT cells. We also observed that kinesin and K40 α‐tubulin were increased in the fractions corresponding to organelles (O), but not in the cytosolic fraction (C) (Figure 6c). These data prompted us to analyse the specific binding of kinesin‐1 to mitochondria, which allows cells to move mitochondria towards the cell periphery. We isolated mitochondria (M) from siCtrl and siCCT cells and found that kinesin‐1 was more enriched in mitochondrial fractions from siCCT cells (Figure 6d). This might explain the decreased distance between the mitochondria and the plasma membrane in these cells (Figure 4f). We also found that peroxisomes are consistently more distant from the centrosome in siCCT cells than in mock‐transfected and siCtrl cells (Figure 6e,f and Figure S5). The number of peroxisomes per volume and the distance from the centrosome to peroxisomes are similar in mock‐transfected and siCtrl cells (Figure 6f), suggesting that these organelles could also be relocated towards the periphery in siCCT cells, similar to mitochondria. Taken together, these data indicate that CCT plays an essential role in the organization of the cell, which affects the number and quality of inter‐organelle contacts, thereby promoting deep changes in cellular metabolism.

## 4 DISCUSSION

The activity of the chaperonin CCT is essential for the correct function of a growing number of proteins, due to its folding‐assisting activity (Willison, 2018). It is also relevant to control the subcellular positioning of proteins through lateral interactions (Hodeify et al., 2018). The diminished availability of CCT in cells causes a decrease in autophagy levels, which limits the cell ability to degrade aggregated proteins due to misfolding (Pavel et al., 2016). However, there are additional active pathways that allow the cell to deal with excess of aggregated proteins, such as their sorting and release through the formation of ILVs and their secretion as EVs (Vidal et al., 1997). In this regard, the involvement of CCT in the dynamics and biogenesis of the ELS is an attractive hypothesis that we have addressed in the present study through loss‐of‐CCT‐function approaches. Our findings identify a specific lipid signature in cells with reduced CCT (siCCT cells), which results in the decrease in LDs and lysosomes that is accompanied by an increment in peroxisomes and a deflected ELS pathway directed to biogenesis of ILVs and their subsequent release as EVs.

Our data show that CCT is a common component of EVs released by human Jurkat cells and mouse T lymphoblasts, suggesting a relation between the chaperonin and the ELS pathway. The study of the proteomic profile of human Jurkat cells and their secreted EVs evidenced specific changes in CCT‐silenced (siCCT) cells, which display a differential protein signature showing increase of proteins related to molecular transport in EVs. Since EVs can be used to sort specific proteins to be released by the cell (Maas et al., 2017; van Niel et al., 2018), these results suggest the intrinsic implication of CCT in proteostasis through this mechanism, apart from others that include collaboration with co‐chaperones such as prefoldin, described in previous reports (Gestaut et al., 2019). Furthermore, IPA assessment of the proteomic profile of siCCT cells and their released EVs suggest changes in lipid metabolism. Accordingly, our data from a high‐throughput lipidomic approach demonstrate that siCCT cells has a specific lipid profile, with lower FAs and higher amounts of phospholipids, mono‐, di‐, triglycerides, ceramide and acylcarnitine. This reveals that CCT have a direct role in lipid metabolism regulation. This role could be exerted indirectly through the folding of mLS8 subunit, among others pertaining to mTORC1 and 2, performed by CCT (Cuéllar et al., 2019). In particular, we hypothesize that mTORC1 dysregulation caused by limited amount of CCT could cause an increment of lipid biogenesis, since mTORC1 has been described as an inhibitor of SREBPs and PPARγ, two transcription factors that induce lipogenesis and adipogenesis (Peterson et al., 2011; Settembre et al., 2013). In some cell systems, there is an ESCRT‐independent formation of ILVs in MVB that requires ceramide (Trajkovic et al., 2008) and/or protein aggregation (Vidal et al., 1997). These results, together with the specific increment of ceramide and other well described EV‐enriched lipids such as LPC, LPE, PC, PG, PE, MG, DG and TG in siCCT cells, provided us a clue to focus our investigation on the study of the ELS in CCT silenced cells. Indeed, the increase in TG together with the reduction in LD size and number in siCCT cells may alter the TG‐phospholipid balance in the plasma membrane, which is found in tumour cells (Pakkanen et al., 2011). The presence of TG islands in the cell membrane is related to events of curvature and scission, since TG are not able to form lipid bilayers (Pakkanen et al., 2011). This may also be related to the increased biogenesis of EVs in siCCT cells, since it would help the scission of the limiting bilayer in the late endosomes to form ILVs. Other proposed controllers of ILV biogenesis are proteins belonging to the tetraspanin family, such as CD81 and CD63 (Charrin et al., 2009), which are selectively enriched in the ILVs of MVB and in their derived EVs (Simons & Raposo, 2009). CD63 is predominantly enriched intracellularly, localizing to late endosomes and lysosomes in most cell types (Pols & Klumperman, 2009). CD63 is also present in lysosome‐related organelles, such as neutrophil myeloperoxidase granules and endothelial cell Weibel‐Palade bodies (Pols & Klumperman, 2009; Raposo & Marks, 2007). Our data show that the number of EEA1^+^ particles decreases in siCCT cells, while its MFI remains unaltered. In parallel, CD63^+^ particles are increased in these cells while CD63 MFI decreases. These results indicate that the dynamics of the ELS favours the biogenesis of MVB when CCT is a limiting factor. This was confirmed by cryoSXT and confocal microscopies. The increase in MVB leads to an increased production of ILVs, which are released as EVs, as demonstrated through NTA and biochemical analyses. Another plausible explanation for the increased EV production in siCCT cells could be the intrinsic dysregulation of proteostasis. The reduced availability of CCT may cause a considerable amount of misfolded and non‐functional proteins that could be extremely difficult to manage through classical pathways (Martin‐Cofreces et al., 2021; Willison, 2018). Proteins that are not properly folded in cells can be subjected to a number of processes. They can be ubiquitinated and targeted for proteasomal degradation. Alternatively, these proteins can be directed to the autophagic pathway (Moreno‐Gonzalo et al., 2018). Thus, aberrant proteins are captured by autophagosomes, delivered to MVBs, and degraded upon fusion of those vesicles with lysosomes. Taking into account the findings showing an association between a reduced activity of CCT with autophagy defects (Pavel et al., 2016; Tang et al., 2021) and lysosome malfunction (Martin‐Cofreces et al., 2020; Pavel et al., 2016), it is conceivable that siCCT cells use other pathways for the clearance of these proteins. Whilst other chaperones such as Hsp70 are considered to be more general chaperones, CCT has a rather restricted range of protein clients (Yam et al., 2008). In fact, some of these novel CCT substrates could be directly involved in lipid metabolism and regulate directly or indirectly the endolysosomal pathway, such as mTORC1 and C2 subunits (Cuéllar et al., 2019). mTORC1 is an inhibitor of autophagy through the phosphorylation of ULK1 at serine‐757 and serine‐473, which significantly decreases its activity and its interaction with AMPK (Kim et al., 2011). Recently, a signalling pathway downstream mTOR that regulates CCT interaction with methylases METTL3 and METTL14 has also been related to autophagy inhibition. These methylases introduce the N6‐methyladenosine modification in RNAs, which is decreased by mTORC and CCT inhibition and/or silencing, facilitating the degradation of autophagy‐related transcripts (Tang et al., 2021). In addition to the release of cargos to the lysosomes, the MVBs can promote protein clearance by favouring the biogenesis and secretion of EVs (Vidal et al., 1997). Unfolded proteins are deflected towards ILVs, which are then released to the extracellular medium encapsulated into EVs. This may also explain the alterations observed in the lipid profile of cells, which could be reprogrammed as a survival mechanism to favour EV biogenesis. We conclude that CCT could be a key modulator of protein processing. Thus, an unusual low expression of this chaperonin causes a misbalance in the cell, which guides the general protein destination towards MVB pathway instead of to autophagy processing and lysosome degradation.

Since FAs were reduced in siCCT cells, we decided to focus on those organelles that are responsible for their storage and degradation. First, we demonstrated by both cryoSXT and confocal microscopy that LDs have their number and radius reduced. This effect correlates with a tendency to decrease the neutral lipid levels in siCCT cells detected with a specific fluorescent probe, albeit not significantly. This contrasts with the increased amount of TG found in these cells, but relates to the specific reduction of cytoplasmic FAs found in the foregoing lipidomics data. We also found a reduced number of lysosomes in siCCT cells. Interestingly, contacts between LDs and lysosomes increased in these cells, whereas mitochondria‐LD contacts decreased. These results suggest that there is direct delivery of lipids from LDs to lysosomes. Indeed, it has been previously suggested that CCT is required for maintaining lysosome function to allow autophagy and that this is due to defects in the actin cytoskeleton (Pavel et al., 2016). Since actin is a major client for CCT (Llorca et al., 2001, 1999; Yam et al., 2008), this can be an attractive hypothesis. However, our results point to additional regulation of the biogenesis of organelles and the control of their contacts, which might also be dependent on cytoskeleton organization. In this sense, tubulins are also major clients of CCT (Llorca et al., 2001, 1999; Willison, 2018). Our research supports that CCT is regulating organelle positioning through changes in post‐translational modifications of cytoskeleton components, resulting in differences in the proportion of motor proteins such as kinesin‐1, physically linked to organelles such as mitochondria. Our confocal microscopy data demonstrate that peroxisomes increase both their number and volume in siCCT cells. Moreover, peroxidised lipids are also increased in these cells. The increased number of peroxisomes may arise from the decreased rate of autophagy described for siCCT cells (Pavel et al., 2016), which is used by the cell to eliminate peroxisomes (Klionsky et al., 2007). Taken together, these results suggest that FAs enter inside peroxisomes, probably through direct contact with LDs (Binns et al., 2006), where they can be metabolized through FAO. Our results show that acylcarnitine, the lipid responsible of the transit of FA between peroxisomes and mitochondria, is increased in siCCT cells. Moreover, our Seahorse analyses show that mitochondria from siCCT cells reduce their respiration rate when glucose is the energy source whereas they rise it when using palmitate. In addition, glycolysis rate increases in siCCT cells, supporting a strong implication of CCT in the regulation of cell metabolism. Since both glycolysis and FAO converge into acetyl‐coA, we postulate that the acetylation state can be altered in siCCT cells. The increment in acetylated microtubules in siCCT cells may be consequence of this metabolic status. Kinesin‐1 binds preferentially to acetylated microtubules, which would promote its activity. Kinesin‐1‐driven anterograde movement of organelles may explain the anomalous positioning of important metabolic organelles such as LDs, mitochondria and peroxisomes as well as their ability to interact with each other and with other organelles. Also, peroxisome positioning can be changed due to alterations in their biogenesis from mitochondria and therefore indirectly dependent on kinesin‐1 (Fransen et al., 2017). These changes in intracellular organization may explain the metabolic shift observed in homeostasis.

On the other hand, our proteomic approach points to a correlation between CCT silencing, DNA/RNA damage processes and lipid pathways such as arachidonic acid or thromboxane. Both histone acetylation and the products of lipid peroxidation are related to DNA damage. An example is the peroxidation of arachidonic acid, which leads to 4‐dydroxy‐2‐nonenal (HNE) formation. HNE has been proven to react with DNA and proteins to form adducts that are implicated in certain human pathologies such as cancer (Douki et al., 2004; Gasparovic et al., 2017; Gentile et al., 2017). Our data indicate a link between CCT functioning and DNA/RNA damage, opening a new perspective in the study of CCT related to maintenance of cellular homeostasis. It is known that CCT helps the folding of some checkpoint proteins for the control of cell cycle (Martín‐Cófreces et al., 2021). However, cell viability is not affected by the extent of silencing in this system, as observed previously (Martin‐Cofreces et al., 2020). Cancer cells over‐express different subunits of the chaperonin, such as CCT1 and CCT2, which promotes survival and migration (Guest et al., 2015). Indeed, CCT expression is known to be reduced with age, which would lead to specific cell defects, such as lower rate of division and protein aggregation (Klaips et al., 2018). Further research is needed to elucidate the role of this chaperonin in processes whose products are described to be related to human pathologies due to DNA damage and ROS generation. For example, it would open a new perspective in the investigation of cancer processes based on chaperone functioning. Interestingly, CCT malfunctions have been related to age‐associated proteostasis defects in some neurodegenerative pathologies (Behrends et al., 2006; Brehme et al., 2014; Kitamura et al., 2006; Tam et al., 2006). This suggests that defective cells may have a decrease or a lack of CCT function, provoking an internal homeostasis dysregulation. To overcome this problem, it is thus conceivable that CCT knock‐down cells could promote EV release to eliminate misfolded proteins. The possible link between CCT defects and these disorders is an open question for future research.

## AUTHOR CONTRIBUTIONS

Author order of Amelia Rojas‐Gomez and Sara G. Dosil was randomly assigned. Amelia Rojas‐Gomez, experimental design, data curation (molecular and cell biology and transfection/nucleofection, Seahorse assays, lipidomics, proteomics, Western blot), image composition, writing (original draft, review and editing), Figures 2, 4l–q, 5h–j, Figure S1, 4, 5; Sara G. Dosil, experimental design and data curation (molecular and cell biology and transfection/nucleofection, fluorometry, microscopy, proteomics, Western blot. qRT‐PCR), image composition, writing (original draft, review and editing), Figure 1, 4, 5h–j, Figure S1 and S3; Francisco J. Chichón, conceptualization, resources, funding acquisition, data curation (SXT and correlative microscopy) Figure 3a–c, Figure 5f–g, Figure S2; Nieves Fernandez‐Gallego, proteomics data curation and analysis Table 1; Alessia Ferrarini and Rocio Tarifa, lipidomic analysis Figure 2; Enrique Calvo, proteomic analysis, Figure 1, Table 1; Diego Calzada‐Fraile, performed experiments (cytometry, microscopy Bodipy experiments) Figures 3, 4; Silvia Requena and Montserrat Arroyo, microscopy, western blot, Figure 4, 6; Joaquin Oton, SXT data curation Figure 3a–c, Figure 5f–g; Andrea Sorrentino and Eva Pereiro, SXT resources; Andrea Sorrentino, Seahorse statistical analysis Figure 4l–q, Figure S4; Jesus Vazquez, proteomic analysis, data curation, writing (original draft) and funding acquisition; José Valpuesta, conceptualization, resources, funding acquisition, writing (original draft); Francisco Sanchez‐Madrid planned and coordinated research, discussed results, and supervised and contributed to manuscript writing; Noa Martín‐Cófreces planned and coordinated research, conceptualization, resources, funding acquisition, data curation, analysed and interpreted data, performed experiments (Figures 3 to 5a–g, 6, Figure S2–S5), and wrote the manuscript with input from all authors.

## CONFLICT OF INTEREST STATEMENT

The authors declare no conflicts of interest.

## Supporting information

Supporting Information

Supporting Information

Supporting Information

Supporting Information

Supporting Information

Supporting Information

Video

Video

## Data Availability

All data will be available through public repositories or by asking the corresponding author. The lipidomic data can be accessed directly via its Project DOI: https://doi.org/10.21228/M8SH87. This study is available at the NIH Common Fund's National Metabolomics Data Repository (NMDR) website, the Metabolomics Workbench, https://www.metabolomicsworkbench.org where it has been assigned Study ID ST002185. This work is supported by NIH grant U2C‐DK119886. The mass spectrometry proteomics data have been deposited to the ProteomeXchange Consortium via the PRIDE partner repository with the dataset identifier PXD034917.
