# Supplementary figures and images for: Chaperonin CCT controls extracellular vesicle production and cell metabolism through kinesin dynamics
Source: J Extracell Vesicles. 2023 Jun 16;12(6):12333. doi: 10.1002/jev2.12333 (PMC10276179; doi:10.1002/jev2.12333)

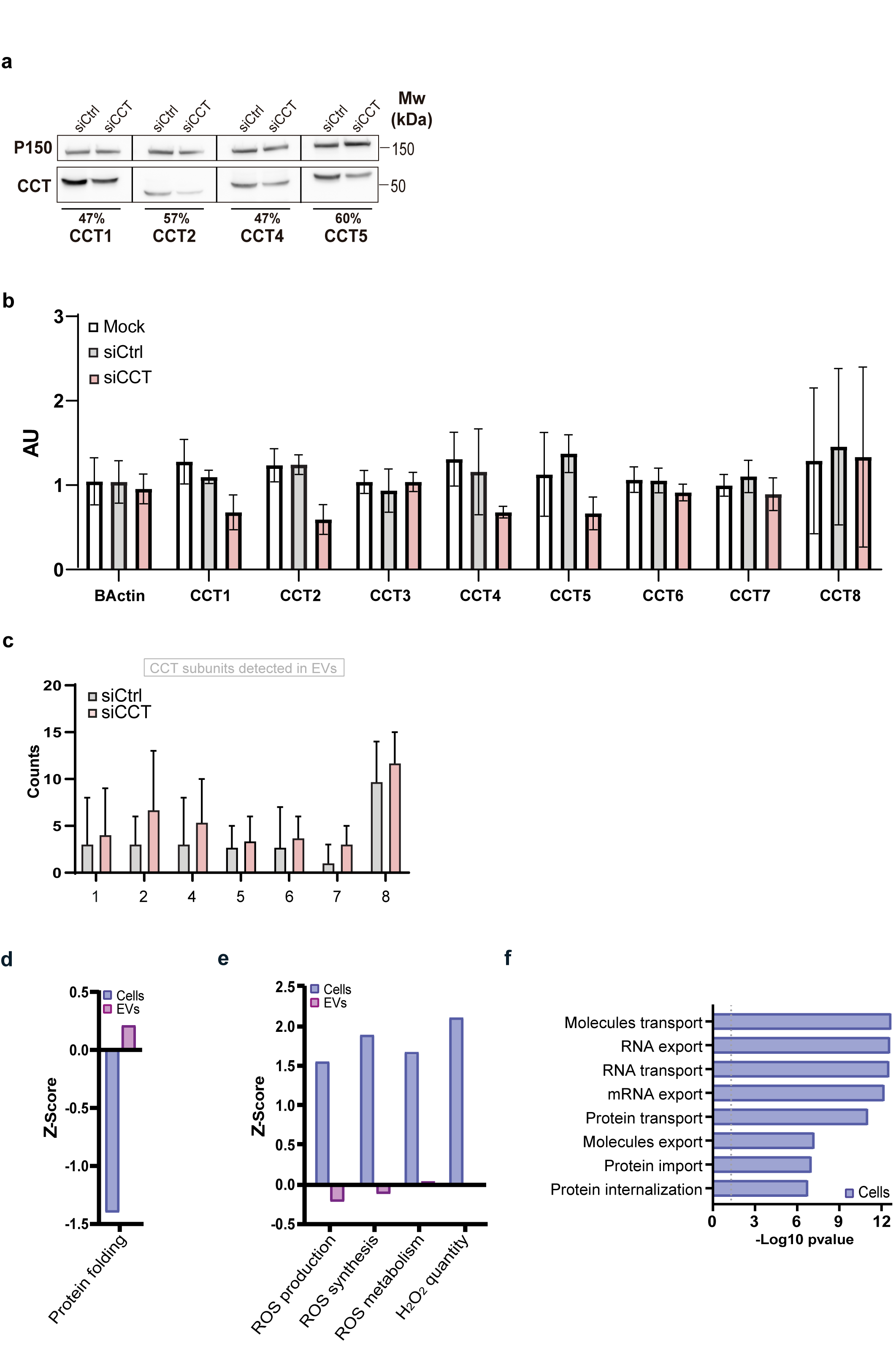

Supplement: Supplementary file 1 — Supporting Information [file JEV2-12-12333-s003.tif]

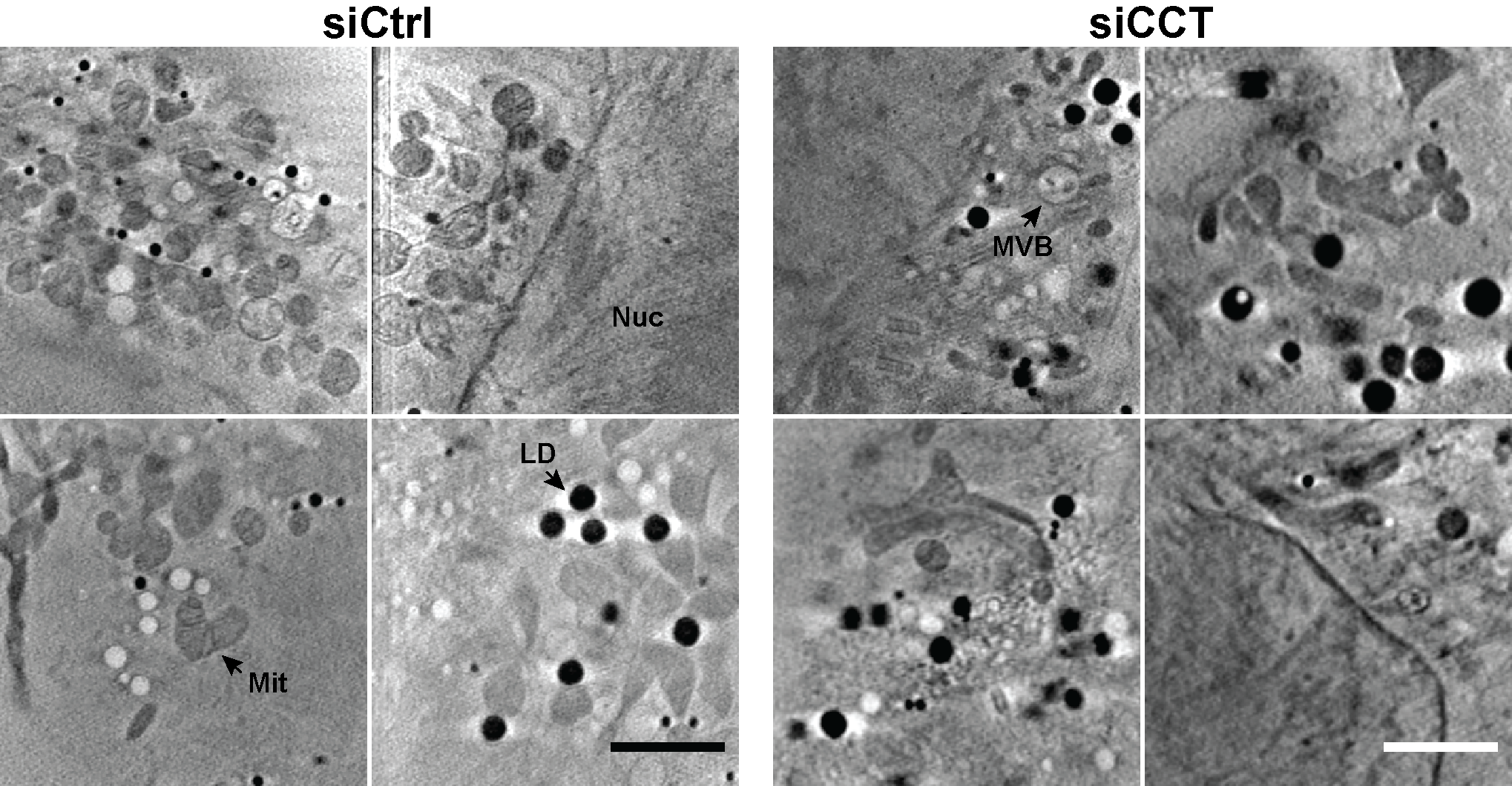

Supplement: Supplementary file 2 — Supporting Information [file JEV2-12-12333-s002.tif]

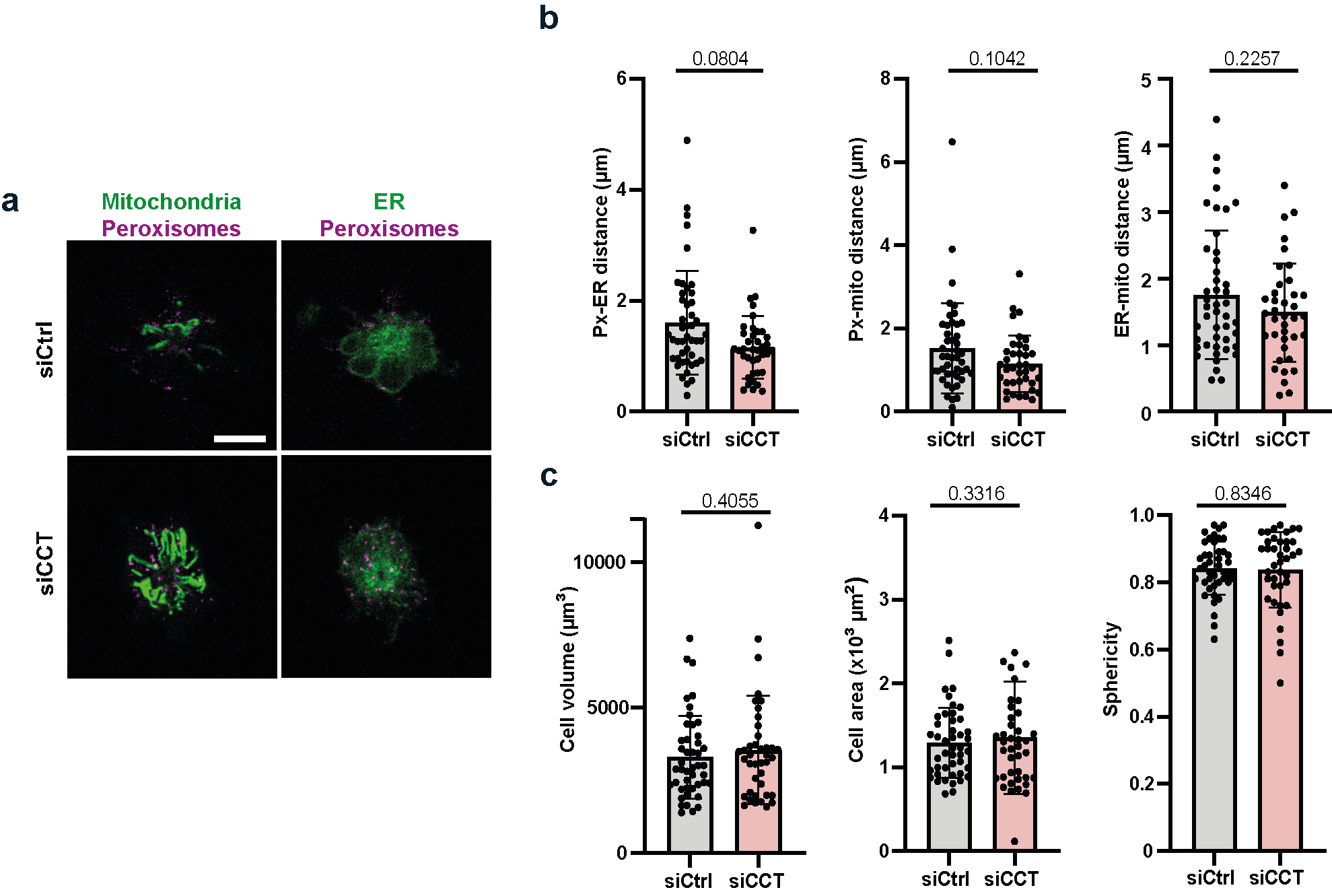

Supplement: Supplementary file 3 — Supporting Information [file JEV2-12-12333-s008.tif]

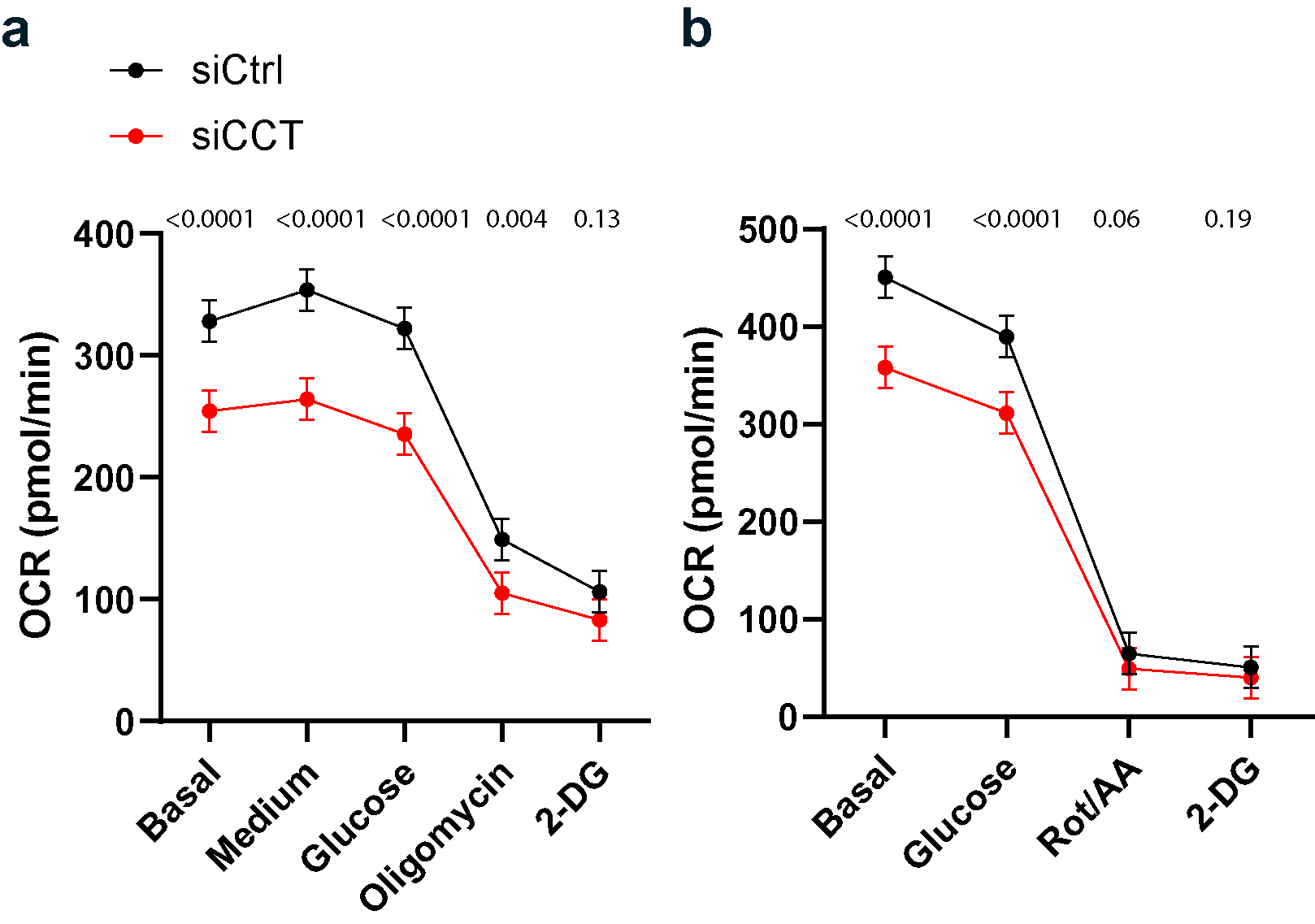

Supplement: Supplementary file 4 — Supporting Information [file JEV2-12-12333-s006.tif]

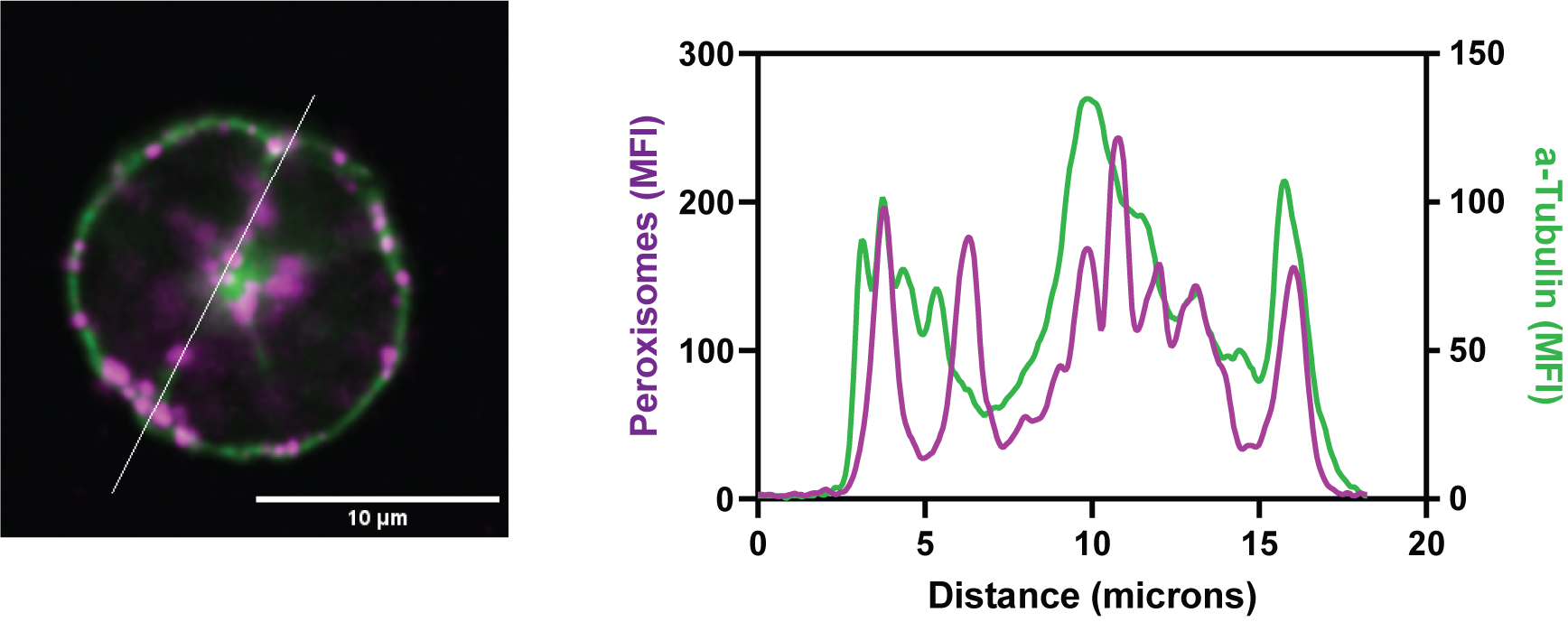

Supplement: Supplementary file 5 — Supporting Information [file JEV2-12-12333-s005.tif]
