# Supplementary material for: Chaperonin CCT controls extracellular vesicle production and cell metabolism through kinesin dynamics
Source: J Extracell Vesicles. 2023 Jun 16;12(6):12333. doi: 10.1002/jev2.12333 (PMC10276179; doi:10.1002/jev2.12333)
Supplement: Supplementary file 6 — Supporting Information [file JEV2-12-12333-s001.docx]

| **Metabolite abbreviated name** | **Biochemical main class** |
| --- | --- |
| Cer 42:2;O2 | Ceramide |
| PC 35:3 // PE 38:3 | Glycerophospholipids |
| DG 38:1 | Diacylglycerol |
| MG 20:1 | Monoacylglycerol |
| TG 49:1 | Triacylglycerol |
| TG 48:1 | Triacylglycerol |
| CAR 18:0 | Carnitine |
| AA-5HT | N-acyl amines |
| LPC 20:4 | Lysophosphatidylcholine |
| LPC 22:6 | Lysophosphatidylcholine |
| PC O-36:5 | Phosphatidylcholine |
| PC O-35:5 | Phosphatidylcholine |
| PG 36:0 | Phosphatidylglycerol |
| DG 36:1 | Diacylglycerol |
| PC 34:2 | Phosphatidylcholine |
| PG 38:0 | Phosphatidylglycerol |
| DG 32:5 | Diacylglycerol |
| LPE 18:3 | Lysophosphatidylethanolamine |
| PE 38:4 | Phosphatidylethanolamine |
| PC O-32:2 | Phosphatidylcholine |
| Epoxy-dihydroxy-norvitamin D3 | Vitamin D3 |
| DG dO-30:0 | Diacylglycerol |
| TG 48:2 | Triacylglycerol |
| LXD4 | Eicosanoids |
| TG 50:2 | Triacylglycerol |
| TG 52:2 | Triacylglycerol |
| DG 46:7 | Diacylglycerol |
| DG 46:4 | Diacylglycerol |
| Acyl-capnine | Sphingolipids |
| FA 20:0 | Fatty Acids |
| Ianosteryl 18:1//Campestenyl 20:2 | Sterols |
| PE 23:2;O2 | Oxidized glycerophosphoethanolamines |
| PE 24:2;O3 | Oxidized glycerophosphoethanolamines |
| FA 12:3 | Fatty Acids |
| FA 16:4;O | Fatty Acids |
| Oxo-PGE1 | Prostaglandins |
| LPG 14:0 | Lysophosphatidylglycerol |
| FA 14:3 | Fatty Acids |
| FA 20:4;O3 | Fatty Acids |
| FA 22:10 | Fatty Acids |
| PG 21:1;O | Oxidized glycerophosphates |
| LPG 20:0 | Lysophosphatidylglycerol |
| SM 38:2;O2 | Sphingomyelins |
| FA 22:4;O4 | Fatty Acids |
| FA 22:7 | Fatty Acids |
| ST 24:1;O5;GlcA | Bile Acids |
| FA 26:1;O | Fatty Acids |
| FA 16:0;O | Fatty Acids |
| FA 18:0;O2 | Fatty Acids |

**Supplementary Table 1.** Abbreviations for lipid metabolites found in the study.

| **Gene** | **RefSeq** | **Forward Primer** | **Reverse Primer** |
| --- | --- | --- | --- |
| CCT1/TCP1 | NM_030752.3 | GGAAACGATCCGCTCCCAAA | CCAAGCCAACTGGACCAAGA |
| CCT2 | NM_006431.3 | AGAGGTGATTCTGCGTGTGG | ACAGCACGTGGGAATGCTTA |
| CCT3 | NM_005998.5 | TGGAAACATCAATGCTGCCAAG | GCCTCCCATTGGGTCCAAAA |
| CCT4 | NM_006430.4 | AGTATTACATCCAGCAGCCAGAA | GGTGCCATCTCCTGCTTCTA |
| CCT5 | NM_012073.5 | ACCGCAAGTCCCGTCTTATG | ATTTGCTACAGCCTTTGCTGC |
| CCT6A | NM_001762.4 | GGCAATGTGCTGCTTCACG | ATCATCCTGGGCTGTTGCTA |
| CCT7 | NM_006429.4 | GGCGGGCCGCTGAATAA | TGGGAGCTATCAGTCCCCTC |
| CCT8 | NM_006585.4 | CAGATGCTCAAGGAGGGAGC | TGTACGAGTGGTTTGGGCAAG |
| HPRT1 | NC_000023.11 | CCTGGCGTCGTGATTAGTGAT | AGACGTTCAGTCCTGTCCATAA |
| ACTB | NM_001101.5 | ATCATGTTTGAGACCTTCAA | AGATGGGCACAGTGTGGGT |

**Supplementary Table 2.** Primers for RT-qPCR.
